# Safety and efficacy of phosphodiesterase-5 (PDE-5) inhibitors in fetal growth restriction: a systematic literature review and meta-analysis

**DOI:** 10.3389/jpps.2024.13206

**Published:** 2024-08-15

**Authors:** Ying Liu, Ella Man-Wai Un, Ying Bai, Man Keong Chan, Luo Xin Zeng, Sut Leng Lei, Junjun Li, Carolina Oi Lam Ung

**Affiliations:** ^1^ Department of Pharmacy, Kiang Wu Hospital, Macau, Macao SAR, China; ^2^ Institute of Chinese Medical Sciences, University of Macau, Macau, Macao SAR, China; ^3^ Department of Public Health and Medicinal Administration, Faculty of Health Sciences, University of Macau, Macau, Macao SAR, China

**Keywords:** fetal growth restriction, phosphodiesterase-5 inhibitors, sildenafil, tadalafil, intrauterine growth restriction

## Abstract

**Introduction:** Fetal growth restriction (FGR) is associated with a higher risk of perinatal morbidity and mortality, as well as long-term health issues in newborns. Currently, there is no effective medicine for FGR. Phosphodiesterase-5 (PDE-5) inhibitors have been shown in pre-clinical studies to improve FGR. This study aimed to evaluate the latest evidence about the clinical outcomes and safety of PDE-5 inhibitors for the management of FGR.

**Methods:** Eight databases (PubMed, Embase, Medline, Web of Science, Cochrane Library, Chinese National Knowledge Infrastructure, Chinese Biomedical Database and WangFang Database) were searched for English and Chinese articles published from the database inception to December 2023. Randomized controlled trials (RCTs) reporting the use of PDE-5 inhibitors in FGR were included. The quality of the RCTs was assessed using the Cochrane Risk of Bias Tool. Odds ratio and mean difference (MD) (95% confidence intervals) were pooled for meta-analysis.

**Results:** From 253 retrieved publications, 16 studies involving 1,492 pregnant women met the inclusion criteria. Only sildenafil (15 RCTs) and tadalafil (1 RCT) were studied for FGR. Compared with the control group (placebo, no treatment, or other medication therapies), sildenafil increased birth weight, pregnancy prolongation and umbilical artery pulsatility indices. However, it also increased the risk of pulmonary hypertension in newborns, as well as headache and flushing/rash in mothers. There were no significant differences in gestation age, perinatal mortality or major neonatal morbidity, stillbirth, neonate death, infants admitted to neonatal intensive care unit, intraventricular hemorrhage and necrotizing enterocolitis in infants, as well as pregnancy hypertension and gastrointestinal side effects in mothers between the treatment and the control groups.

**Discussion:** Sildenafil was the most investigated PDE-5 inhibitors for FGR. Current evidence suggests that sildenafil can improve birth weight and duration of pregnancy but at the same time increase the risk of neonatal pulmonary hypertension. It remains uncertain whether the benefits of sildenafil in FGR outweigh the risks and further high-quality RCTs are warranted.

**Systematic Review Registration:** https://www.crd.york.ac.uk/prospero/display_record.php?RecordID=325909

## Introduction

Fetal growth restriction (FGR), also known as intrauterine growth restriction (IUGR), is one of the most common pregnancy complications, affecting 5–10% of all pregnancies [1]. It is defined as a fetus that does not reach its genetic potential for growth and development due to pathological factors, characterized by estimated fetal weight (EFW) or abdominal circumference less than the 10th percentile for gestational age [2]. FGR is associated with a higher risk of perinatal morbidity and mortality, as well as long-term health issues such as impaired neurological and cognitive development and cardiovascular or endocrine diseases in adulthood [1]. Currently, there is a lack of effective therapy available for FGR, maternal nutritional supplementation [3], bed rest [4], oxygen therapy [5], aspirin [6], and low molecular weight heparin (LMWH) [7] have all been studied as the treatment for FGR, but the evidence for their benefits in the normal course of FGR is limited. Fetal health monitoring to determine the best delivery timing remains the main management strategy [8].

The etiology of FGR is complex, with the most common risk factors being suboptimal uterine-placental perfusion and fetal nutrition [8]. Normal fetal growth requires the exchange of nutrients and substrates at the maternal-fetal interface, which is enabled by the uteroplacental and umbilicoplacental circulations. The reduction of uteroplacental blood flow and the resultant hypoperfusion in the placenta might increase reactive oxygen species formation, which would in turn impair vasodilation through altering nitric oxide (NO) generation [9]. The trophoblast produces NO throughout pregnancy, which dilates the blood vessels of the fetal placental circulation; however, in situations of FGR, a decrease in NO release during pregnancy has been reported [10].

Phosphodiesterase-5 (PDE-5) is a cyclic guanosine monophosphate (cGMP) metabolizing enzyme. The selective inhibition of PDE-5 can increase cGMP and hence increase the bioavailability of NO, which promotes the relaxation of vascular smooth muscle and uterine placental blood flow [11]. PDE-5 inhibitors have been shown in pre-clinical studies to improve FGR by increasing vasodilation and blood flow in the uteroplacental circulation [12–15]. The majority of research tested the clinical effect of sildenafil, although alternative medicines such as the longer-acting tadalafil have also been investigated.

Prior to this review, at least 7 similar systematic reviews had already been published. The review by Chen J *et al.* [16] analyzed 10 studies which compared L-arginine and sildenafil with placebo on FGR. However, only one study used sildenafil, which limited the ability to draw a reliable comparative conclusion. The review by Paauw N.D *et al.* [12] analyzed 22 studies which investigated the effects of sildenafil in pregnancy and found that sildenafil increased fetal growth. However, significant variations across the included studies were observed as both human and animal studies were included for analysis. Another review by Dunn L *et al.* [17] also focused on sildenafil in pregnancy and found that there were no severe adverse effects for the mother nor the newborn. As noted by the authors, a limitation of the review was the inclusion of case reports, case series, and small cohort studies, in addition to only 3 relatively small RCTs which lacked consistent and uniform reporting standards. The review by Ferreira R *et al.* [18] analyzed 7 studies to evaluate the effects of sildenafil in pregnancy, with a focus on FGR in only 4 of these studies. The conclusion drawn from this review was that sildenafil led to improvements in fetal birth weight, reductions in UA-PI, and an increase in maternal headaches. However, the limitations of this review were the small study population for FGR and the high heterogeneity among the included studies.

In another review conducted by Hessami K *et al.* [9], the focus was on PDE-5 inhibitors in FGR pregnancies and included 7 studies. This review revealed that PDE-5 inhibitors might enhance uteroplacental, but not fetal cerebral blood perfusion. However, this review solely focused on uteroplacental and fetal cerebral perfusion, without reporting other clinical effects or safety outcomes. The analysis by Turner J.M *et al.* [19] analyzed 10 studies to evaluate the safety of PDE-5 inhibitors during pregnancy with a specific focus on FGR in only 6 of the included studies. The findings suggested that although there might be mild maternal side effects, prolonged use for the treatment of FGR could potentially increase the risk of pulmonary hypertension in neonates. However, this review encompassed all pregnancy disorders resulting from uteroplacental insufficiency, exhibiting a wide range of manifestations and likely differing pathophysiology, and thus possibly increased heterogeneity. The review by Rakhanove Y *et al.* [20] analyzed 9 studies which compared the effects of sildenafil on FGR and concluded that sildenafil was associated with increased birth weight and prolonged pregnancies while showing no significant effect on neonates. However, the effects of uteroplacental and fetal cerebral perfusion were not reported. Furthermore, this study lacked detailed data regarding the effects of prolonged pregnancies. In light of the gaps in the existing findings, a more comprehensive report regarding the efficacy and safety of PDE-5 inhibitors on pregnancies with FGR is warranted.

Furthermore, according to the previous reviews [9, 12, 16–18, 20], PDE-5 inhibitors could increase fetal growth and improve uteroplacental blood flow, while posing minimal safety concerns for both mother and fetus. Nevertheless, the benefits of PDE-5 inhibition in FGR remain controversial, as Hessami K *et al.* [9] mentioned no difference between PDE-5 inhibitors versus placebo on prolongation of pregnancy or improved pregnancy outcomes. Additionally, Turner J.M *et al.* [19] revealed that PDE-5 inhibitors had no effect on gestation at birth and the sildenafil group had a higher risk of neonatal pulmonary hypertension when compared to the placebo group, with the result being strongly influenced by the Dutch STRIDER trial [21]. Concerning the safety and the limited possibility of significant benefits, the Dutch and Canadian STRIDER trials were halted before the recruitment targets were completed [22]. The safety of PDE-5 inhibitors is worth further analysis.

Considering the limitations of previous reviews in terms of the small study number, the inclusion of various study designs, the discrepancies in study objectives, the incompleteness of reporting of efficacy and safety of PDE-5 inhibitors on FGR, as well as the inconclusive findings about the efficacy and safety of PDE-5 inhibitors on FGR, a comprehensive and systematic literature review and meta-analysis is considered necessary. As such, this study aims to systematically evaluate the most recent evidence about the efficacy and safety of PDE-5 inhibitors for FGR to better support decision-making in clinical practice.

## Methods

This systematic review and meta-analysis were conducted according to the PRISMA 2020 Statement and inclusive of the 27-item checklist [23]. The study protocol has been registered in the PROSPERO international prospective register of systematic reviews (Registration number: CRD42022325909).

### Search strategy and study selection

The literature search was performed in five English databases (including PubMed, Embase, Medline, Web of Science, and the Cochrane Library) and three Chinese databases (including Chinese National Knowledge Infrastructure (CNKI), Chinese Biomedical Database (CBM), WangFang Database). The search period was set from database inception to December 2023. The combination of the two concepts: “fetal growth restriction” AND “phosphodiesterase 5 inhibitors” and the related terminologies (such as “fetal growth retardation,” “Intrauterine Growth Retardation,” etc., for the first concept, and “PDE5 Inhibitor,” “sildenafil,” “vardenafil”, etc. for the second concept) were used to formulate the search strategy.

To identify appropriate keywords, in addition to Medical Subject Headings (MeSH) terms, popular and commonly used phrases stated in related literature were utilized. The search strategy was developed prior and first conducted in PubMed and CNKI prior to repeating across the other databases. A detailed description of each of the search strategies used in each database is provided in Appendix I.

The studies were checked by two of the authors (YL and EWMU). Following the removal of duplicate publications, the remaining studies were screened based on title, abstract, inclusion, and full-text by authors YL and EWMU. In order to confirm the quality and consistency of the screening procedure, the screening findings were compared and examined to see if there was any discrepancy between the decisions made by YL and EWMU. Agreement on inclusion and proposal for exclusion according to the predefined inclusion and exclusion criteria were confirmed with the third author (COLU).

### Inclusion and exclusion criteria

Studies written in English or Chinese and published in peer-reviewed journals that reported the results of randomized controlled clinical trials (RCTs) investigating the use of PDE-5 inhibitors for the treatment of FGR were included in this review. Non-RCT studies, conference abstracts, book chapters, editorials, dissertations, and case reports were excluded. The comparators in the RCTs may be placebo, no treatment, or other medication therapies.

The primary efficacy outcomes measures were birth weight (grams), pregnancy prolongation after randomization and, gestational age at birth. The safety outcomes included adverse effects on the neonate and mother, including the perinatal mortality/major neonatal morbidity, stillbirth, neonate death, admitted to NICU, intraventricular hemorrhage (IVH), Necrotizing enterocolitis and pulmonary hypertension of infants, as well as headache, flushing/rash, Gastrointestinal side effects, and pregnancy hypertension in mothers. The secondary outcomes include uterine artery pulsatility indices (UtA-PI), umbilical artery pulsatility indices (UA-PI), and middle cerebral artery pulsatility indices (MCA-PI).

### Data extraction

Data was extracted independently by two of the authors (SLL and LXZ) and will be retrieved and entered into an Excel spreadsheet (the first author’s name, the country of the study, the year of publication, the study design, the inclusion criteria of FGR, the population’s characteristics, the number and age of the patients, the type of PDE-5 inhibitor used, the dose of medication and duration of treatment, the combination therapy, the comparators, the primary and secondary outcomes).

### Risk of bias assessment

YB and MKC independently assessed the quality of the randomized controlled trials using the Cochrane Risk of Bias Tool (RoB 2) [24].

### Data synthesis

The birth weight, pregnancy prolongation after randomization and, gestation age at birth, UA-PI, MCA-PI, UtA-PI were represented by the mean difference (MD) and 95% confidence interval (CI), while the odd ratios (OR) and 95% CI were used to represent the perinatal mortality or major neonatal morbidity, stillbirth, neonate death, infants admitted to NICU, IVH, necrotizing enterocolitis, pulmonary hypertension, headache, flushing/rash, gastrointestinal side effects, pregnancy hypertension.

We used the random-effect model and calculated the *I* ^2^ to assess the heterogeneity across the studies included for analysis. The *I* ^2^ of 25%, 50%, and 75% represented low, medium, and high heterogeneity respectively. The source of heterogeneity was explored via subgroup analysis based on the maternal age (under or above 30 years old) [25]. All statistical analyses were performed with Review Manager Version 5.3. Original data expressed as median or interquartile range were used to estimate the sample mean and standard deviation for analysis in this study [26].

## Results

### Study selection

**FIGURE 1:**
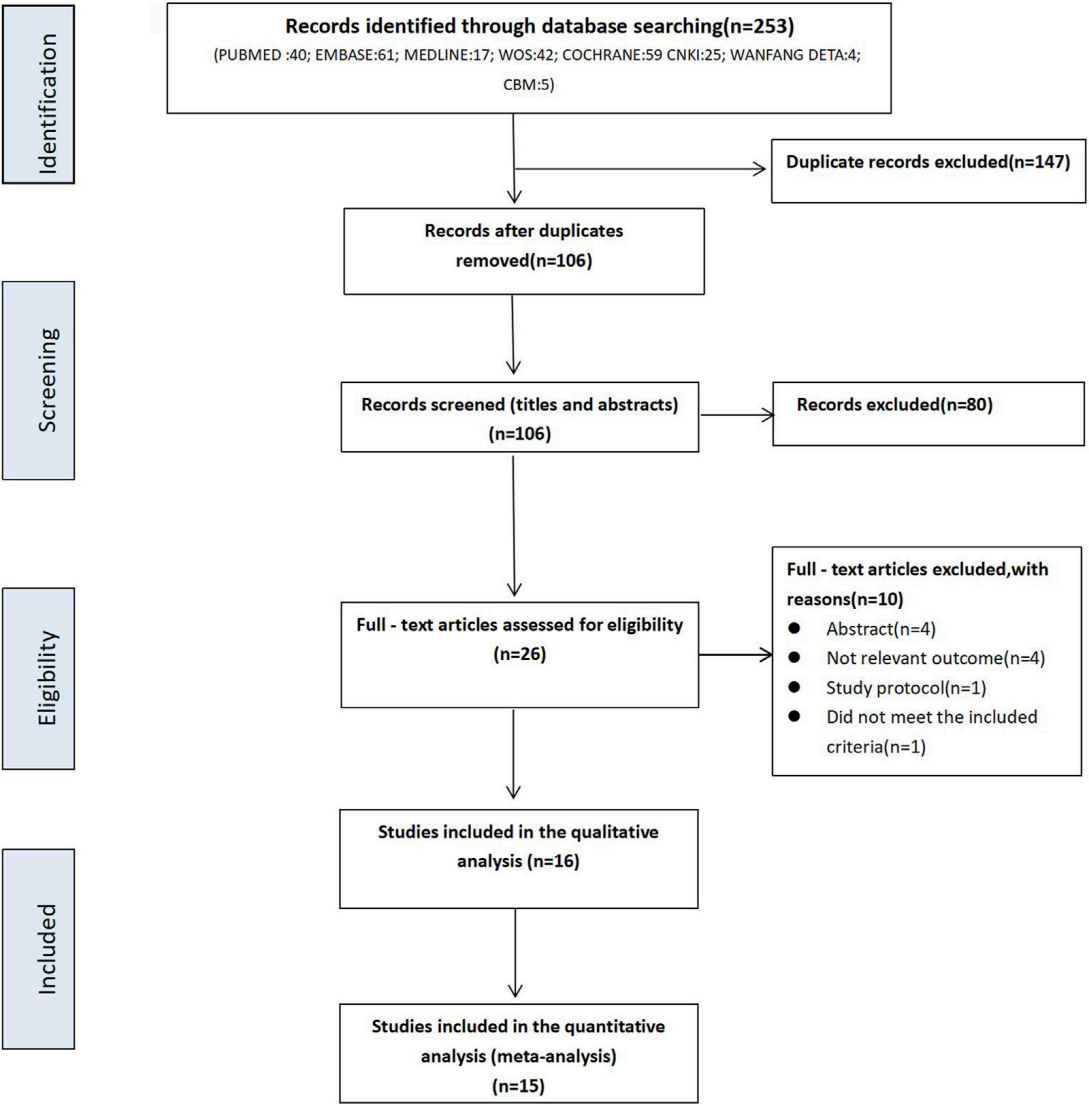
PRISMA Flowchart of literature search.

A total of 253 articles were identified through an initial search in the databases including PubMed (n = 40), Embase (n = 61), Medline (n = 17), Web of Science (n = 42), Cochrane Library (n = 59), CNKI (n = 25), WanFang Data (n = 4), CBM (n = 5). After the removal of duplicates, 106 articles remained. Subsequently, 80 records were excluded by reviewing the title and abstract while 26 full-text articles were retrieved to assess their eligibility in terms of the inclusion criteria. Based on this assessment, 10 studies were excluded due to the following reasons: 4 were abstract only; 4 did not report relevant outcomes; 1 was study protocol, and 1 did not meet the inclusive criteria. Ultimately, 16 relevant trials were included for qualitative analysis as shown in Figure 1. Considering 15 trials investigated sildenafil and only 1 evaluated **tadalafil**, only the 15 trials investigating the effect of sildenafil were included for the meta-anlaysis.

### Studies characteristics

The demographic characteristics of the included studies are reported in Table 1. Studies were published between 2012 and 2022. Five studies were conducted in Egypt [27–31], 2 in India [32, 33], 2 in Iran [34, 35], 1 in Brazil [36], 1 in China [37], 1 in Japan [38], 1 in the Netherlands [21], 1 in New Zealand and Australia [39], 1 in the UK [40] and 1 in Canada [41].

**TABLE 1:** Characteristics of the included studies.

	Study ID	Country	Inclusion criteria of IUGR fetuses	Intervention			Number of patients (control or comparators group/treatment group)	Mean maternal age (control or comparators group/treatment group)	Mean gestational age (control or comparators group/treatment group)	Duration of treatment
				Treatment group	Combination therapy (if suitable)	Control or comparators group				
1	A. Abdelshafy et al. 2019	Egypt	Fetal weight below 10th percentile for GA	Sildenafil citrate 25 mg every 8 h orally	NA	Placebo orally	90 (45/45)	27.35/26.76	NA	Until delivery
2	A. Pels et al. 2020	Netherlands	Fetal weight below 5th percentile for GA	Sildenafil 25 mg three times a day orally	N/A	Placebo orally	215 (107/108)	31.00/31.00	24.70^b^/24.61^b^	Until fetal death, 32 weeks of gestation, or birth
3	A. Sharp *et al*. 2018	UK	Fetal weight below 10th percentile for GA	Sildenafil 25 mg three times a day orally	The exposure to antenatal corticosteroids and magnesium sulphate given for neuroprotection was similar in both groups	Placebo orally	135 (65/70)	32.85^a^/29.14^a^	25.62^b^/25.10^c^	Until 32 weeks and 0 days' gestation or delivery
4	A. T. Jr et al. 2016	Brazil (Santa Catarina)	Fetal weight below 10th percentile for GA	Sildenafil 50 mg orally once daily	N/A	Placebo orally	24 (12/12)	25.80/23.60	30.10/28.50	N/A
5	K. Groom *et al.* 2019	New Zealand and Australia	At 22^+0^–27^+6^ weeks of gestation if the fetal abdominal circumference was ≤3rd centile and at 28^+0^–29^+6^ weeks of gestation if the estimated fetal weight1was <700 g	Sildenafil 25 mg three times a day orally	N/A	Placebo orally	122 (59/63)	31.40/31.40	24.80/24.50	Until 32^+0^ weeks of gestation, birth delivery or fetal death (whichever occurred first)
6	M. A. El-Sayed et al. 2017	Egypt	Abnormal Doppler indices	Sildenafil 50 mg orally once daily	N/A	Placebo orally	54 (27/27)	28.15/26.30	29.39/29.74	Till delivery
7	M. A. El-Sayed *et al.* 2018	Egypt	Abdominal circumference <5th centile, or Doppler umbilical artery (UA) pulsatility index (PI) > 95th centile	Sildenafil citrate 50 mg orally, one single dose	N/A	Placebo orally	54 (27/27)	28.15/26.30	31.81/32.04	Single dose
8	M. V. Dastjerdi et al. 2012	Iran	The percentage of sonographic estimate was within 3% of actual birth weight	Sildenafil citrate 50 mg orally, one single dose	N/A	Placebo orally	41 (27/14)	32.00/25.64	35.00/35.00	Single dose
9	N. A. A. Shehata *et al.* 2018	Egypt	Singleton pregnancy at gestational age 24–34 weeks, abdominal circumference (AC) <5th percentile with an estimated probability of intact survival of <50%	Sildenafil citrate 20 mg three times a day orally	Oral fish oil 150 mg syrup twice daily and oral zinc capsules 25 mg once daily; All participants received betamethasone for lung maturation (two doses of 12 mg, 24 h apart) at enrollment in the study	Oral placebo similar to sildenafil in addition to fish oil and zinc supplementation	46 (23/23)	30.70/30.40	30.10/29.50	Until delivery or fetal demise (whichever comes first)
10	N. Eshraghi et al. 2021	Iran	Fetal weight below 10th percentile for GA	Sildenafil 25 mg orally once daily	N/A	Placebo orally	80 (40/40)	29.85/31.88	34.04/34.10	N/A
11	Q. Tonggang *et al.* 2021	China	Fetal weight below 10th percentile for GA	Sildenafil 25 mg three times a day orally	LMWH(1 mL:2500IU) 1 mL sc BID. High protein and high energy diet	LMWH(1 mL:2500IU) 1 mL sc BID with high protein and high energy diet	116 (58/58)	30.62/31.98	25.56/24.67	28 days
12	R. Singh *et al.* 2018	India	Fetal weight below 10th percentile for GA	Sildenafil citrate 25 mg three times a day orally	N/A	L-arginine 3 mg twice a day	218 (108/110)	NA	NA	Until delivery
13	R. Yadav *et al.* 2021	India	Fetal weight below 10th percentile for GA	Sildenafil citrate 25 mg three times a day orally	Injection betamethasone was given to all patients who delivered before 34 weeks or who were planned for elective lower segment Cesarean section at any gestational age	N/A	130 (65/65)	25.80/26.06	26.80/26.48	Until delivery
14	S. Maki et al. 2019	Japan	Fetal growth less than 1.5 standard deviations below the mean estimated fetal body weight, based on the Japanese standard	Tadalafil 20 mg orally once daily	Conventional treatment for FGR according to Japanese guidelines	Conventional treatment for FGR according to Japanese guidelines	87 (43/44)	32.23^/32.94^	27.82^/28.65^	Until delivery
15	Z. Sanad et al. 2019	Egypt	Fetal weight below 10th percentile for GA	Sildenafil citrate 50 mg orally, one single dose	N/A	Placebo orally	60 (30/30)	26.70/25.97	29.83/30.30	Single dose
16	P. v. Dadelszen *et al.* 2022	Canada	estimated fetal weight<700 g and fetal AC<10th percentile for GA	Sildenafil 25 mg three times a day orally	N/A	Placebo orally	20 (9/11)	30.00/33.50	22.57^b^/21.71^b^	until either delivery or 31 + 6 weeks

^a^ Original data expressed as median were used to estimate the sample mean and standard deviation [26].

^b^ Original data expressed as interquartile range were used to estimate the sample mean and standard deviation [26].

^c^ Original data expressed as an interquartile range that were significantly skewed away from normality and thus the normal-based methods were not applied for data transformation.

The population of the included trials consisted of 1,492 pregnant women, with 747 and 745 pregnancies being in the treatment group and control/comparators group respectively. The gestational age of the initial treatment ranged from 22 to 35 weeks. The minimum and maximum dosage of sildenafil used across the included studies ranged from 25 to 75 mg daily and the dosage of tadalafil was 20 mg once daily. Treatment duration ranged from single-dose before Doppler assessment in 3 studies [28, 31, 35], up to delivery or fetal death in 10 studies [21, 27, 29, 30, 32, 33, 38–41], up to 28 days in 1 study [37]or was not mentioned in 2 studies [34, 36].

Sildenafil citrate was used as the treatment group, while placebo was used as the control/comparators group in 10 trials [21, 28–31, 34–36, 39, 41] and L-arginine as the control/comparators group in 1 trial [33]. One study [27]used sildenafil citrate in addition to fish oil and zinc supplementation as the treatment group to compared with placebo in addition to the same supplementation as the control/comparators group. One study [40]used sildenafil citrate in addition to injection of corticosteroids and magnesium sulphate as the treatment group, and placebo in addition to the same injection as the control/comparators group. One study [32] used sildenafil citrate in addition to injection of betamethasone as treatment group, and no intervention as control/comparators group. One trial [37] used sildenafil citrate in addition to LMWH as treatment group, and LMWH as control/comparators group. Tadalafil in addition to conventional treatment for FGR according to Japanese guidelines was used as treatment group, conventional treatment as control/comparators group in 1 trial [38].

Twelve studies reported data on MCA-PI [21, 27–31, 34–36, 38–40], 13 studies on UA-PI [21, 27–31, 33–36, 38–40] and only 2 studies on MAP-PI [28, 36]. The most commonly reported indicators for efficacy were [1]: birth weight (grams) [2]; pregnancy prolongation (in days) [3]; gestation age at birth (weeks) [4]; blood flow (UA-PI and MCA-PI). The most commonly reported indicator for safety were [1]: infants admitted to neonatal intensive care unit (NICU) [2]; headache, flushing/rush, gastrointestinal side effects in mothers [3]; perinatal mortality or major neonate morbidity [4]; intraventricular hemorrhage (IVH), necrotizing enterocolitis in infants.

### Efficacy

#### Birth weight - overall and subgroup analysis (maternal age in experiment group)

Among the included studies in this review, 10 of them had reported the efficacy of PDE-5 inhibitors in FGR in terms of birth weight [21, 27, 29, 30, 32–34, 38–40]. However, only 7 studies were included for further analysis while 3 studies [21, 27, 38] were excluded for the following reasons [1]: The study by Maki *et al.* was the only study that investigated tadalafil and, thus, was excluded in the meta-analysis which focused only on sildenafil in order to minimize heterogeneity [2, 38] The study by Shehata *et al.* reported the birth weight using interquartile range which significantly skewed away from normality distribution, and was thus excluded from further analysis [27]; and [3] The study by Pels *et al.* was halted prematurely due to the safety concerns of sildenafil which prevented the full investigation of efficacy and was thus excluded from the meta-analysis of efficacy [21]. Nevertheless, the findings about the safety of sildenafil reported in this study was included in the subsequent meta-analysis of safety outcome.

**FIGURE 2:**
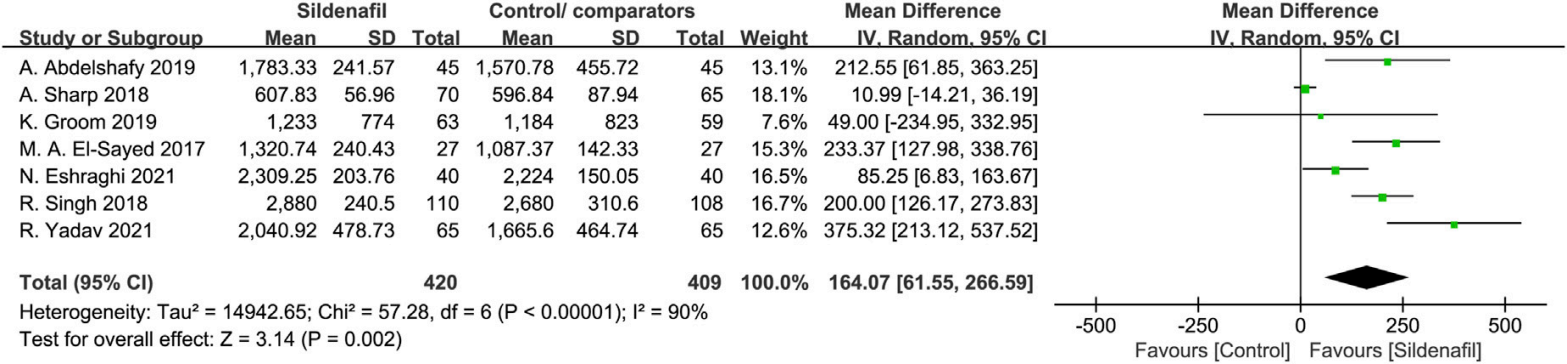
The effect of sildenafil on fetal birth weight (grams)in all mothers.

Fetal birth weight was analyzed in 7 trials [29, 30, 32–34, 39, 40] and included 829 patients (420 in the sildenafil group and 409 in the control/comparators group). Sildenafil was associated with a statistically significant increase of 164.07 g (MD:164.07, 95%CI:61.55–266.59, *P* = 0.002, *I* ^2^ = 90%; Figure 2) in birth weight compared with no sildenafil.

**FIGURE 3:**
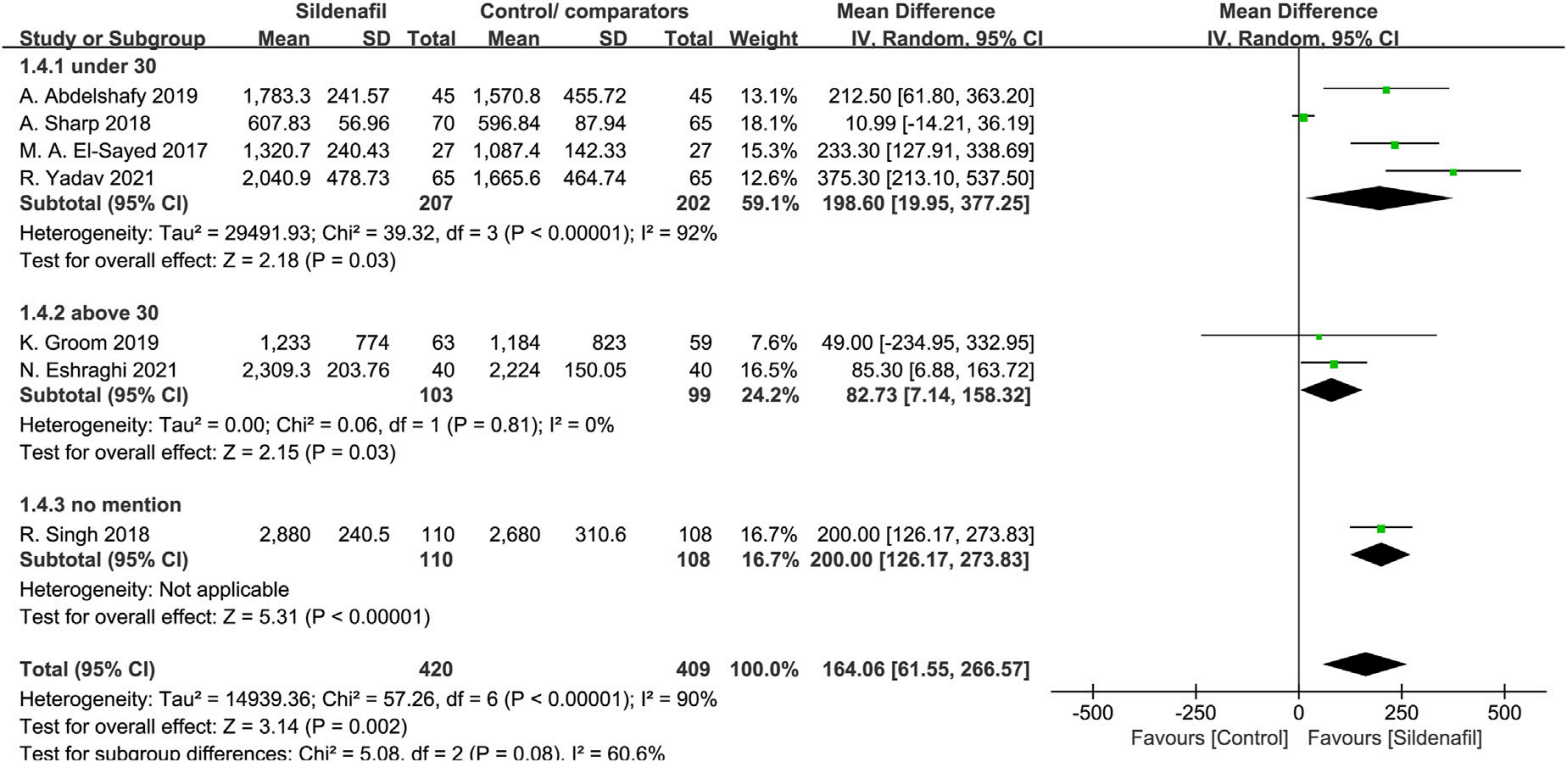
The effect of sildenafil on fetal birth weight (grams) in mothers under or above 30 years old.

Four trials [29, 30, 32, 40], comprising a total of 409 patients, reported on fetal birth weight in mothers under 30 years, and 2 trials [34, 39]were in mothers above 30 years (202 patients). In patients under and above 30 years, sildenafil significantly increases fetal birth weight (MD:198.6, 95%CI:19.95–377.25, *P* = 0.03, *I* ^2^ = 92%; MD:82.73, 95%CI:7.14–158.32, *P* = 0.03, *I* ^2^ = 0%; respectively; Figure 3).

#### Pregnancy prolongation - overall and subgroup analysis (maternal age in experiment group)

**FIGURE 4:**
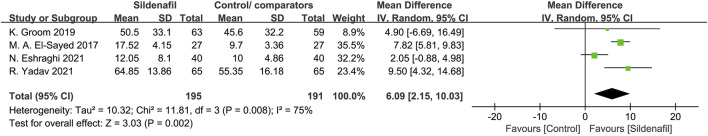
The effect of sildenafil on pregnancy prolongation (days) in all mothers.

Four trials [30, 32, 34, 39] reported on pregnancy prolongation and included 386 pregnant women, 195 in sildenafil group, and 191 in control/comparators group. Sildenafil was associated with a significant increase in pregnancy prolongation for 6.09 days (MD:6.09, 95%CI:2.15–10.03, *P* = 0.002, *I* ^2^ = 75%; Figure 4).

**FIGURE 5:**
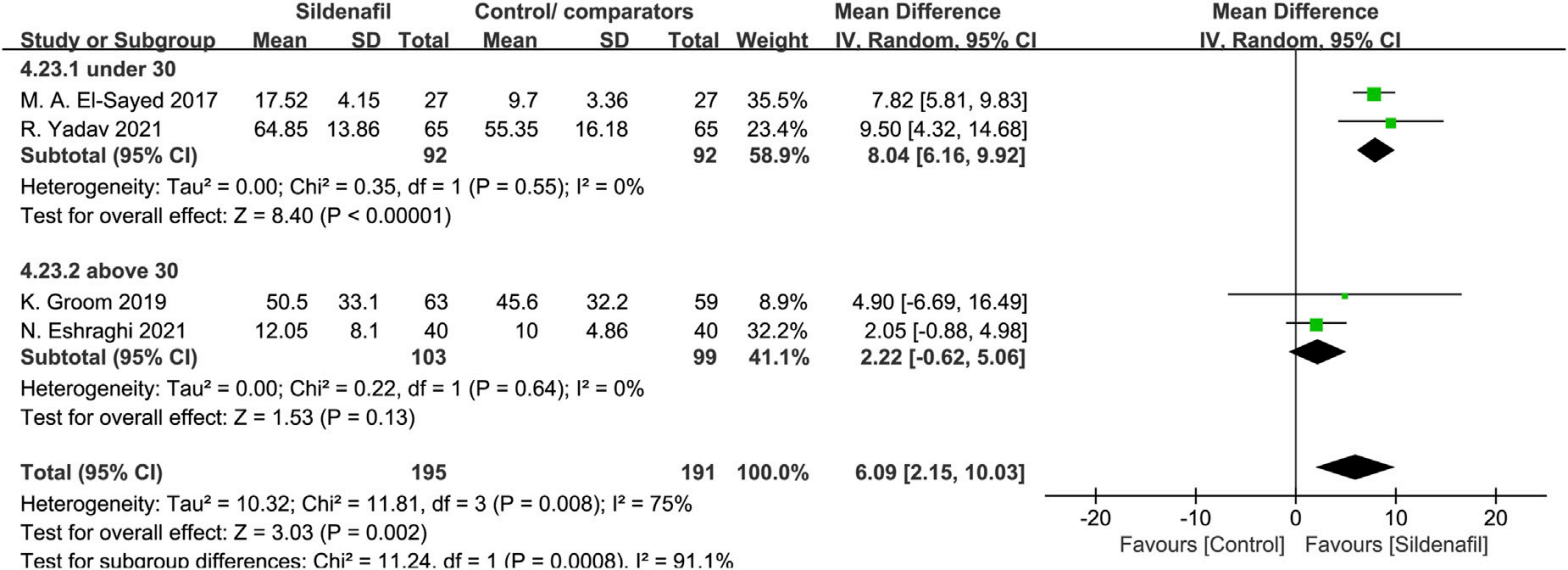
The effect of sildenafil on pregnancy prolongation (days) in mothers under or above 30 years old.

Two trials [30, 32], comprising 184 patients, reported pregnancy prolongation in patients under 30 in the experiment group, 2 trials [34, 39] in patients above 30 (202 patients). Sildenafil-treated pregnant women under 30 showed a significant pregnancy prolongation for 8.04 days (MD:8.04, 95%CI:6.16–9.92, *P* < 0.00001, *I* ^2^ = 0%; Figure 5) compared with control/comparators. In contrast, pregnant women above 30 did not prolong pregnancy in women compared with control/comparators group (MD: 2.22, 95% CI: −0.62 −5.06, *P* = 0.13, *I* ^2^ = 0%; Figure 5).

#### Blood flow—UA-PI

**FIGURE 6:**
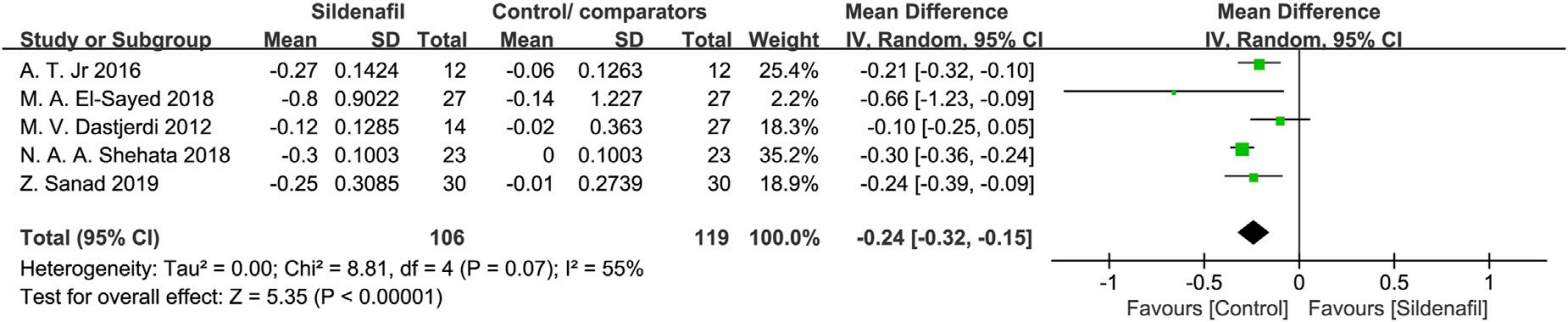
The effect of sildenafil on blood flow-umbilical artery pulsatility indices (UA-PI).

Five studies [27, 28, 31, 35, 36] reported on UA-PI and included 225 pregnant women (106 in sildenafil group and 119 in control/comparators group). Sildenafil was associated with a significant decrease of UA-PI (MD: −0.24, 95%CI: −0.32 – −0.15, *P* < 0.00001, *I*255%; Figure 6).

#### Blood flow—MCA-PI

Five studies [27, 28, 31, 35, 36] reported on MCA-PI and included 225 pregnant women (106 in sildenafil group and 119 in control/comparators group). Sildenafil was not associated with an increase of MCA-PI (MD:0.23, 95%CI: −0.24 −0.70, *P* = 0.35, *I*2 = 98%). No subgroup analysis was conducted in the overall forest plots due to a lack of statistically significant difference.

#### Gestation age at birth

Eight studies [27, 29, 30, 32, 33, 37, 40, 41] reported on gestation age at birth and included 809 pregnant women (409 in the sildenafil group and 400 in the control/comparators group). Sildenafil was not associated with an increase of gestation age at birth (MD: 0.44, 95%CI: −0.29 −1.17, *P* = 0.24, *I* ^2^ = 89%). No subgroup analysis was conducted in the overall forest plots due to a lack of statistically significant difference.

### Safety

#### Infants admitted to NICU

Infants admitted to NICU were reported in 6 studies [27, 30, 33, 34, 39, 40], totaling 593 pregnant women (305 in the sildenafil group and 288 in the control/comparators group). There was no significant difference between the groups in the occurrence of infants admitted to NICU (OR:0.63, 95%CI:0.34–1.19, *P* = 0.16, *I* ^2^ = 56%).

#### Headache in mothers

**FIGURE 7:**
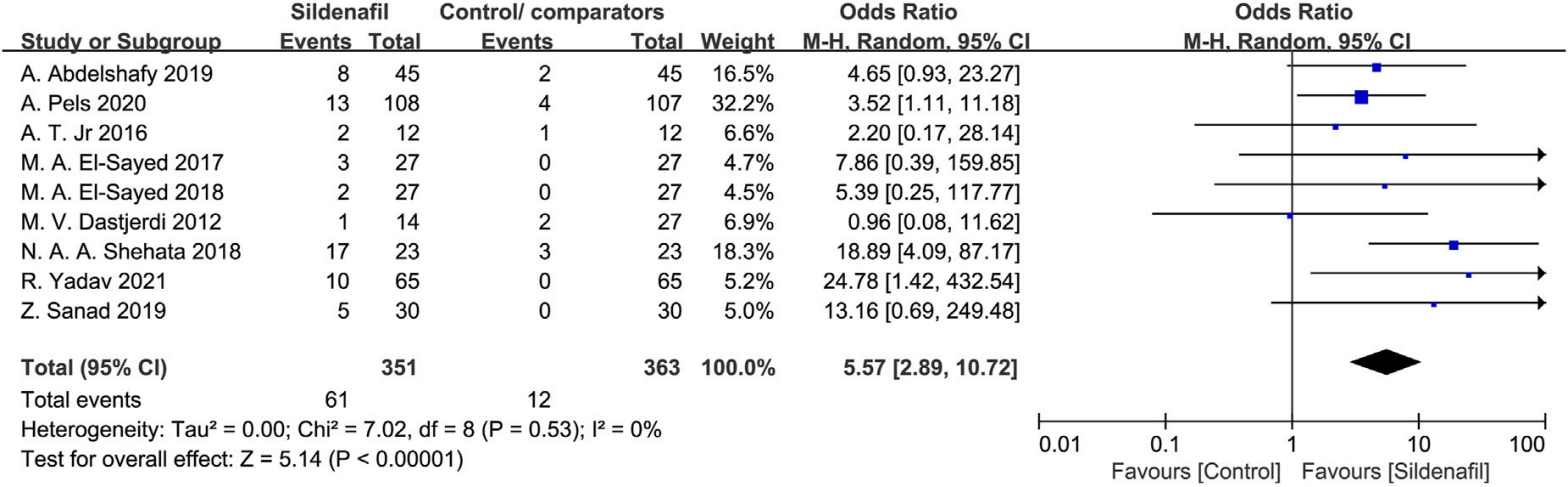
The odd ratio (OR) of headaches in mothers.

Nine studies [21, 27–32, 35, 36]reported on the events of headaches in mothers and included 714 patients (351 in the sildenafil group and 363 in the control/comparators group). Sildenafil was associated with a statistically significant increase in events of headache in mothers (OR:5.57, 95%CI:2.89–10.72, *P* < 0.00001, *I* ^2^ = 0%; Figure 7) compared with no sildenafil treatment.

#### Flushing/rash in mothers

**FIGURE 8:**
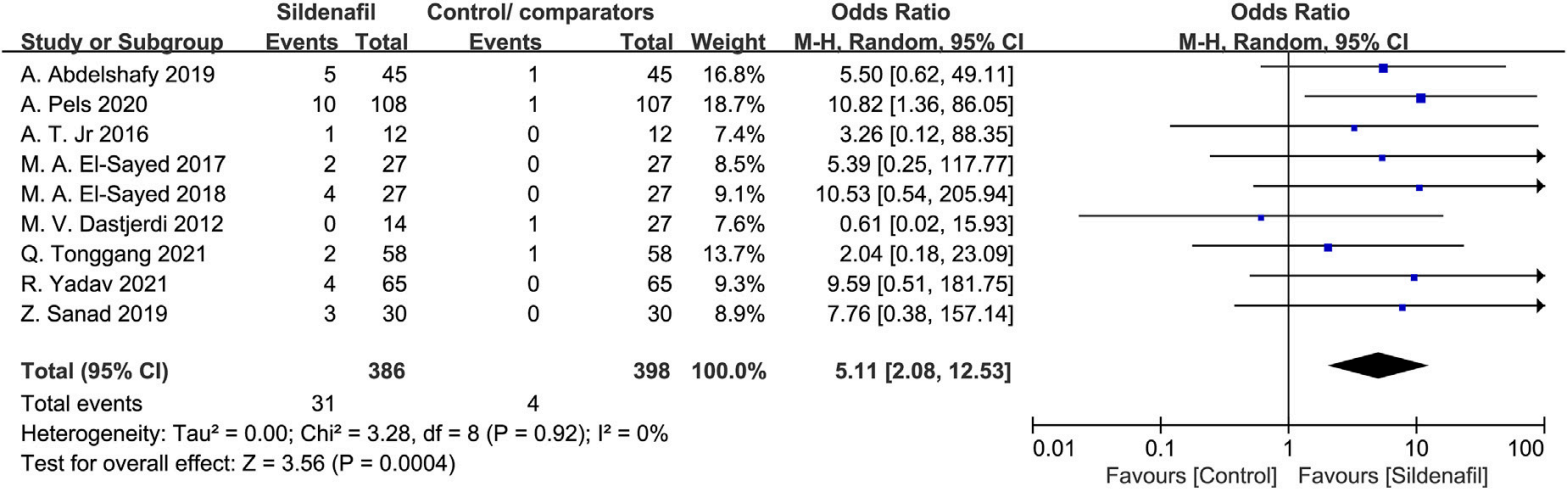
The odd ratio (OR) of flushing/rash in mothers.

Nine studies [21, 28–32, 35–37] reported the events of flushing/rash in mothers and included 784 patients (386 in the sildenafil group and 398 in the control/comparators group). Sildenafil was associated with a statistically significant increase of flushing/rash in mothers (OR:5.11, 95%CI:2.08–12.53, *P* = 0.0004, *I* ^2^ = 0%; Figure 8) compared with no sildenafil treatment.

#### Perinatal mortality or major neonatal morbidity

The number of perinatal mortality or major neonatal morbidity was evaluated in 3 trials [27, 39, 40], with a total of 284 pregnant women (149 in the sildenafil group and 135 in the control/comparators group). Overall, there was no clear difference identified in perinatal mortality or major neonatal morbidity between the control/comparators group or the sildenafil group (OR: 1.02, 95%CI:0.54–1.90, *P* = 0.96, *I* ^2^ = 0%).

#### IVH in infants

Three studies [21, 36, 40] reported on IVH in infants and included 259 patients (136 in the sildenafil group and 123 in the control/comparators group). Overall, there was no clear difference identified in IVH between the control/comparators and sildenafil group (OR:1.46, 95%CI:0.62–3.46, *P* = 0.39, *I* ^2^ = 0%).

#### Necrotizing enterocolitis in infants

Three studies [21, 36, 40] investigated necrotizing enterocolitis in infants and included 279 pregnant women (146 in the sildenafil group and 133 in the control/comparators group). There was no significant difference in events of necrotizing enterocolitis in infants between pregnancy with or without sildenafil treatment (OR:0.60, 95% CI: 0.29–1.23, *P* = 0.16, *I* ^2^ = 0%).

#### Gastrointestinal side effects in mothers

Eight studies [21, 27–32, 37] reported gastrointestinal side effects in mothers and included 765 patients (383 in the sildenafil group and 382 in the control/comparators group). Sildenafil was not associated with a statistically significant increase of gastrointestinal side effects in mothers (OR:1.68; 95%CI: 0.89–3.16; *P* = 0.11, *I* ^2^ = 0%) compared with no sildenafil treatment.

#### Pregnancy hypertension

Five studies [21, 32, 39–41] reported on the events of pregnancy hypertension and included 622 patients (317 in the sildenafil group and 305 in the control/comparators group). Overall, there was no significant difference in the occurrence of pregnancy hypertension between the control/comparators and the sildenafil group (OR:1.11, 95%CI:0.78–1.58, *P* = 0.57, *I* ^2^ = 0%).

#### Stillbirth

The number of stillbirths was evaluated in 3 trials [21, 27, 41], with a total of 281 pregnant women (142 in the sildenafil group and 139 in the control/comparators group). Overall, there was no significant difference identified in perinatal mortality or major neonatal morbidity between the control/comparators group and the sildenafil group (OR:1.24, 95%CI: 0.24–6.41, *P* = 0.79, *I* ^2^ = 52%).

#### Neonate death

Six studies [21, 27, 30, 39–41] reported on neonate death and included 521 pregnant women (272 in the sildenafil group and 249 in the control/comparators group). There was no significant difference in neonate death between pregnancy with or without sildenafil treatment (OR:1.58, 95%CI:0.91–2.76, *P* = 0.11, *I* ^2^ = 0%).

#### Pulmonary hypertension in infants

**FIGURE 9:**
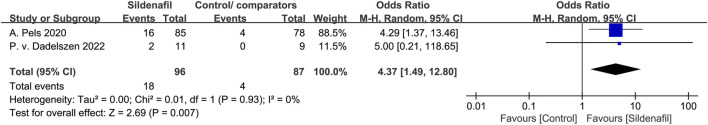
The odd ratio (OR) of pulmonary hypertension in infants.

Two studies [21, 41] reported on the events of pulmonary hypertension in infants and included 183 patients (96 in the sildenafil group and 87 in the control/comparators group). Sildenafil was associated with a statistically significant increase in the events of pulmonary hypertension in infants (OR:4.37, 95%CI: 1.49–12.80, *P* = 0.007, *I* ^2^ = 0%; Figure 9) compared with no sildenafil treatment.

### Study appraisal

**FIGURE 10:**
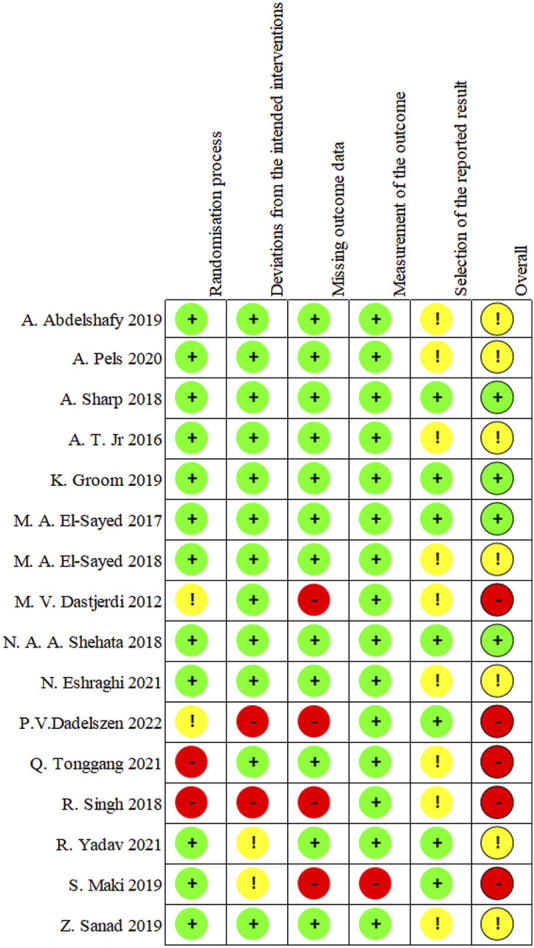
Risk of bias for each study.

**FIGURE 11:**
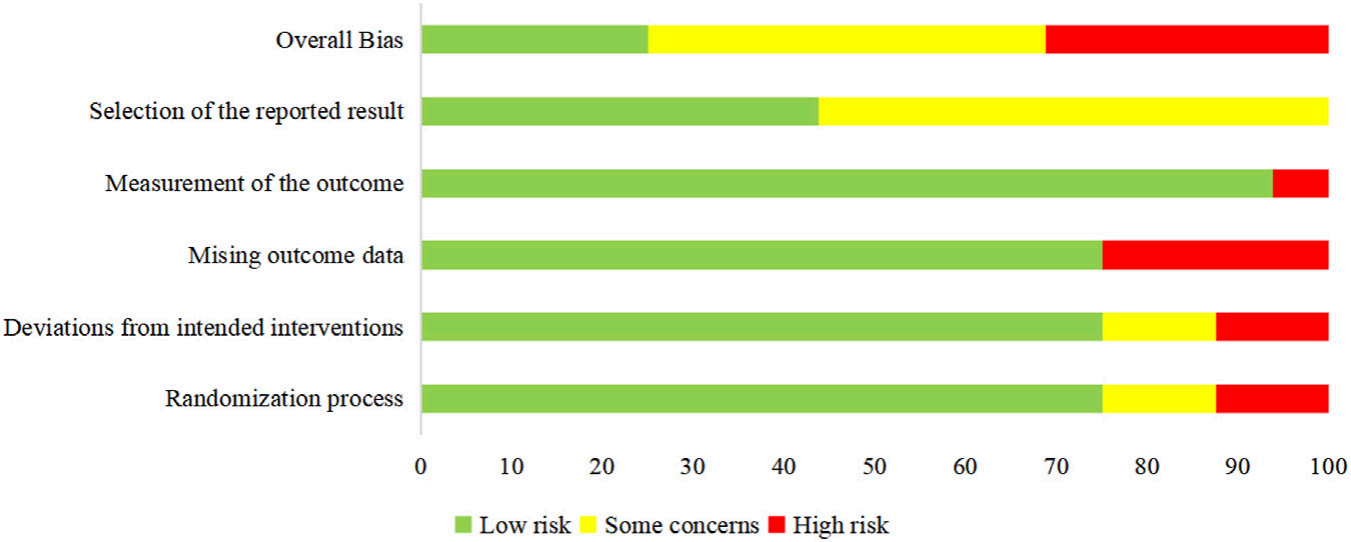
Risk of bias for all studies.

Among the 16 RCTs included in this review, the overall risks of bias (ROB) was recorded as high for 5 trials [33, 35, 37, 38, 41], concerning for 7 of the studies [21, 28, 29, 31, 32, 34, 36], and low for 4 studies [27, 30, 39, 40]. There was no appropriate randomization process in approximately one-fourth of the studies [33, 35, 37, 41]. Bias due to deviations from intended interventions was found in 4 studies [32, 33, 38, 41]. The loss of more than 5% of the sample data occurred in 4 studies [33, 35, 37, 41]. The method of measuring the outcome was inadequately explained in one study [38]. The reported results were selected in around 56% of studies [21, 28, 29, 31–38, 41]. The risk analysis of bias is outlined in Figure 10 which shows the risks of each bias source in each study separately. Figure 11 presents the percentage of each bias source among all the included studies.

## Discussion

This review is based on 16 studies on 1,492 pregnant women treated with PDE-5 inhibitors for FGR compared with placebo, other intervention, or no treatment. In this study, it was found that the treatment with sildenafil for FGR significantly increased birth weight, pregnancy prolongation while significantly decreased UA-PI, but was associated with an increased risk of pulmonary hypertension in new-borns, as well as an increased risk of headache and flushing/rash in mothers. In addition, our results indicated that there were no clinical differences in gestation age, MCA-PI, perinatal mortality or major neonatal morbidity, stillbirth, neonate death, infants admitted to NICU, IVH and necrotizing enterocolitis in infants, as well as pregnancy hypertension and gastrointestinal side effect in mothers. These findings generally align with the conclusions of previous systematic reviews [12, 17–20], except for the one conducted by Chen J *et al.* [16] in which only one RCT was included and no reliable conclusion could be drawn. Nevertheless, in alignment with the recent recommendation made by Groom *et al.* [22], it is concluded in the present review that, considering the higher rate of neonatal pulmonary hypertension, the current evidence does not support the clinic use of sildenafil in FGR management despite the potential benefits in several short-term outcomes. Considering that the current review, as compared to previous ones, included only RCTs for analysis and considered as many clinical outcomes as possible to determine both the efficacy and safety of PDE-5 inhibitors on FGR in larger sample size, the findings being more conclusive could supplement the current evidence base of pharmacotherapy for FGR management.

The major benefit of sildenafil treatment in FGR, according to this meta-analysis, is an increase in fetal weight at birth. A previous study found that low birth weight was not only associated with newborn or infant mortality and morbidity but also related to poorer physical growth, neurodevelopment, and increased risk of chronic metabolic and cardiovascular diseases in adulthood [42]. From this study, the treatment with sildenafil significantly increased fetal birth weight compared with the control group (7 trials, MD: 164.07, 95%CI: 61.55–266.59, *P* = 0.002). In the subgroup analysis by maternal age (under or above 30 years), consistent results were also found. Such results agreed with the previous review [12, 18, 20] which showed that sildenafil could be associated with increased fetal weight at birth. Another prospective study conducted by Maged *et al.* [10] also concluded that the mean birth weight was increased significantly in the sildenafil group than in the no sildenafil group (birth weight was 2066.08 g vs. 1732.8 g). The effect of PDE-5 inhibitors on the uterine vasculature leading to a larger availability of oxygen and nutrients to the growing fetus were believed to be the primary mechanism of action that resulted in the improvement in growth.

Another noteworthy finding from our study was that sildenafil was associated with an overall increase in pregnancy prolongation after enrollment compared with the control group (MD:6.09, 95%CI:2.15–10.03, *P* = 0.002). Our results agreed with the previous review by Rakhanova [20]. However, due to a lack of specific data and forest plots in the previous study, further comparison and interpretation between the two was not feasible. Moreover, different definitions of pregnancy prolongation adopted in the studies also made further comparison of the results from different studies impractical. FGR is associated with an increased risk of premature birth [16], and prolonged duration of pregnancy (gain in intrauterine life) resulting in a lower rate of preterm birth. The guidelines [8, 43, 44] for the management of FGR are likewise based on the benefits of improving fetal maturity by adding some intrauterine days-weeks and avoiding preterm birth. At the same time, the weight of the fetus grows fastest during the third trimester of pregnancy and prolonged latency may also increase birth weight. It is revealed in this study that sildenafil may improve the neonatal birth weight and decrease premature birth. Understandably, these benefits had the value in decreasing admission to the newborn nursery. However, after subgroup analysis, there were no longer any significant differences in pregnancy prolongation among women older than 30 years old. The primary reason is the limited number of existing trials that report this indicator. Future investigation could be designed to focus on determining the beneficial effect of sildenafil on the neonatal birth weight and the decrease in premature birth.

Our findings also demonstrated that sildenafil was associated with a significant decrease in UA-PI (MD: −0.24, 95%CI: −0.32–−0.15, *P* < 0.00001) which were in alignment with findings from previous reviews [9, 18]. The umbilical artery connects the maternal and fetal circulatory systems, and the blood flow status of the umbilical artery can indicate pathological alterations in the placenta. Reference values of the UA-PI gradually decrease during pregnancy, and an increased mean UA PI indicates abnormally high resistance which is a proxy for placental insufficiency [45]. The significant decrease in UA-PI indicates that successful prevention of placental insufficiency is possible. The blood flow of the umbilical artery is raised to meet the substances necessary for fetal growth continuously and to ensure the appropriate development of the fetus [46]. Based on this finding, an improvement in umbilical artery circulation after treatment with sildenafil may lead to an increase in fetal growth.

Safety assessment of PDE-5 inhibitors administration during gestation was another significant outcome measure of this meta-analysis. Our findings indicate that PDE-5 inhibitors did not cause severe maternal side effects, which was consistent with previous systematic reviews [12, 17, 18, 20]. These analyses suggested that PDE-5 inhibitors might be deemed as a safe medicine supported by its potential as a treatment for some maternal and fetal diseases. However, for severe, early onset FGR, we found that sildenafil, when compared with placebo, was associated with a statistically significant increase in neonatal pulmonary hypertension (OR:4.37, 95%CI:1.49–12.80; *P* = 0.007). The study by Turner *et al.* [19] also reported similar risk (RR:2.52, 95%CI:1.00–6.32). Their results were mostly based on the Netherlands trial [21]. Due to a lack of other studies investigating the adverse effect of neonatal pulmonary hypertension, when the study by Pels *et al.* (??) was excluded, the risk for neonatal pulmonary hypertension became insignificant (RR 0.99, 95% CI 0.21–4.78), and the researchers [19] concluded that the adverse outcome was assessed as “moderate certainty.” In our analysis, we newly included the data from the Canadian STRIDER trials [41], and the heterogeneity was low between these studies (*I* ^*2*^ = 0%). Our findings provide additional evidence to substantiate this adverse reaction.

The pathophysiological mechanism underlying the higher rate of pulmonary hypertension in newborns whose mothers were given sildenafil instead of a placebo remains unknown. A possible pathophysiological mechanism could be a “rebound” vasoconstriction after cessation of sildenafil [47]. Even though there is no evidence to support structural changes to the pulmonary vasculature, this cannot be ruled out as a possible mechanism [47]. In addition, a recent 2.5-year follow-up study on children from the STRIDER NZAus Trial revealed that there was no difference in survival without neurosensory impairment between the test group and the placebo group. Nevertheless, it is important to highlight that children who were exposed to sildenafil exhibited a higher likelihood of experiencing cognitive delay [48]. Considering the limited evidence and undetermined potential pathways, it is crucial for future studies to perform long-term monitoring of newborn infants to evaluate their neurodevelopmental outcomes associated with the use of sildenafil in FGR.

Currently, there is only one study on the efficacy of tadalafil and its role in FGR is far from conclusive. In comparison to sildenafil, tadalafil is a selective and long-acting PDE 5 inhibitor and has a faster onset of action [49]. Furthermore, tadalafil and sildenafil may have different mechanisms for fetal-placental perfusion. In a model using human placenta, sildenafil citrate reverses the pre-constricted placental arterial perfusion. However, tadalafil did not show any similar effect [50]. This study indicated that tadalafil might not have passed through the human placental barrier or was degraded by trophoblasts. These findings suggest that there may be variations in the safety and efficacy of sildenafil and tadalafil. Maki *et al.* reported that tadalafil decreased the fetal and infant deaths associated with FGR [38]. In addition, a retrospective study demonstrated that administering tadalafil for FGR might help sustain the increase of fetal head circumference (HC) and improve the neurodevelopmental prognosis of infants [51]. Tadalafil has been suggested to be a choice to manage FGR, but more research is needed to evaluate its efficacy and safety. New high-quality and pragmatic trials comparing different PDE-5 inhibitors treatment for FGR are needed to better inform future clinical decision-making.

### Strengths and limitations

Compared to the previous reviews, our analysis included more clinical outcomes, which gave a more comprehensive report regarding the efficacy and safety of PDE-5 inhibitors on pregnancies with FGR. Firstly, the previous reviews only focused on sildenafil or selected clinical outcomes whereas the current review aimed to analyse all clinical outcome of PDE-5 inhibitors in FGR in terms of efficacy and safety [9, 12, 16–20]. As indicated in the Methods section, a wide range of primary and secondary outcomes were included for analysis which is more comprehensive compared to previous reviews. In addition, the types of studies included in the review also differ. Previous reviews included non-randomized studies and case reports [9, 17], whereas the current review only included RCTs in order to draw evidence-based conclusion. Furthermore, the publication period of the included studies was also the longest in the present review, with an attempt to include all the available RCTs for an up-to-date analysis. Finally, unlike previous reviews which only included literature written in English, the present systematic review searched for literature written in either English or Chinese in both English and Chinese databases.

Our review has some limitations that should be mentioned. Firstly, the high heterogeneity of the included studies may have influenced the dependability of the results even though we used subgroup analysis to control for this variation. However, the probability of residual heterogeneity cannot be ruled out. Secondly, the therapeutic strategy differed greatly across studies, most notably in terms of treatment dosages and duration, although we performed subgroup analyses and found no variation in the results. Furthermore, the mechanism and symptomatology of placental insufficiency syndrome and FGR differ between early and late-onset, however, the potential impact of this distinction was not evaluated in our study. Third, the lack of detailed maternal and infant pharmacokinetic data in all the included studies makes it difficult to speculate on possible reasons for either the lack of effect on most outcomes investigated or the increased risk seen for some complications. All of these factors may contribute to uncertainty biases in the results.

## Conclusion

Sildenafil was the most investigated PDE-5 inhibitors for FGR. The evidence from this review indicates that sildenafil improved birth weight and duration of pregnancy without causing severe maternal side effects. However, it has been shown that sildenafil can increase the risk of neonatal pulmonary hypertension when compared to placebo. It remains uncertain whether the benefits of sildenafil in FGR outweighs the risks and further high-quality randomized clinical trials are warranted. As there was only one trial comparing tadalafil with placebo, the sample size was insufficient to draw reliable conclusions.

## Data Availability

The original contributions presented in the study are included in the article/Supplementary Material, further inquiries can be directed to the corresponding author.

## Abbreviations

FGR, Fetal growth restriction; PDE-5, Phosphodiesterase-5; RCTs, Randomized controlled clinical trials; IUGR, Intrauterine growth restriction; EFW, Estimated fetal weight; NO, Nitric oxide; cGMP, Cyclic guanosine monophosphate; LMWH, Low molecular weight heparin; CBM, Chinese Biomedical Literature Database; CNKI, China National Knowledge Infrastructure; MeSH, Medical Subject Headings; NICU, Neonatal intensive care unit; IVH, Intraventricular haemorrhage; MCA-PI, Middle cerebral artery pulsatility indices; UA-PI, Umbilical Artery pulsatility indices; UtA-PI, Uterine artery pulsatility indices; MD, Mean difference; CI, Confidence interval; OR, Odd ratios; SD, Standard deviation; RR, Risk ratios; ROB, Risk of bias; HC, head circumference.
