# Supplementary figures and images for: Safety and efficacy of phosphodiesterase-5 (PDE-5) inhibitors in fetal growth restriction: a systematic literature review and meta-analysis
Source: J Pharm Pharm Sci. 2024 Aug 15;27:13206. doi: 10.3389/jpps.2024.13206 (PMC11357966; doi:10.3389/jpps.2024.13206)

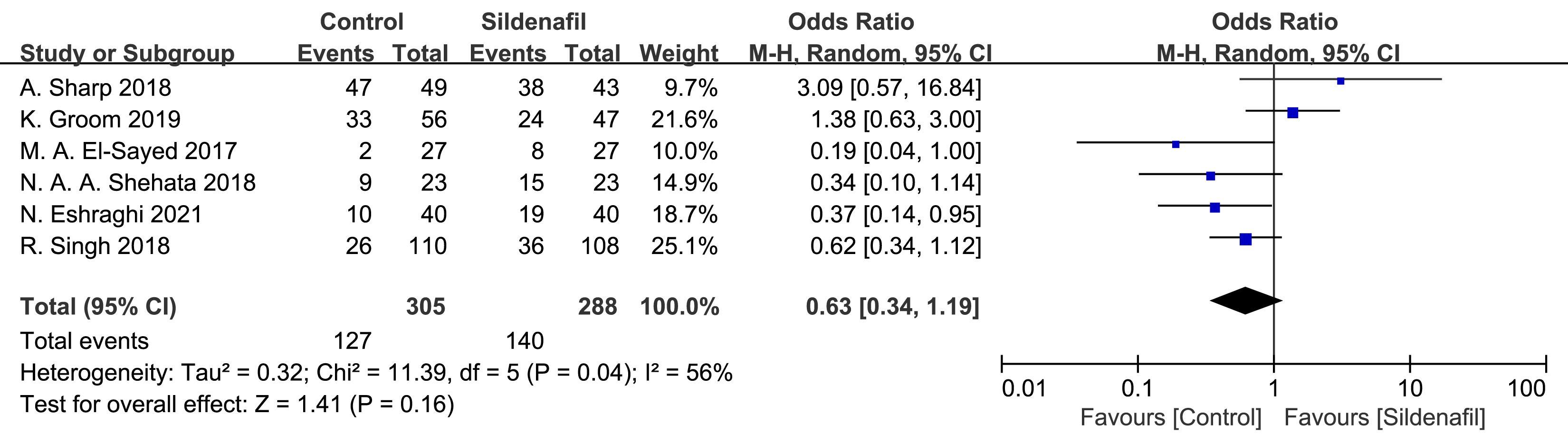

Supplement: Supplementary file 1 [file Image3.JPEG]

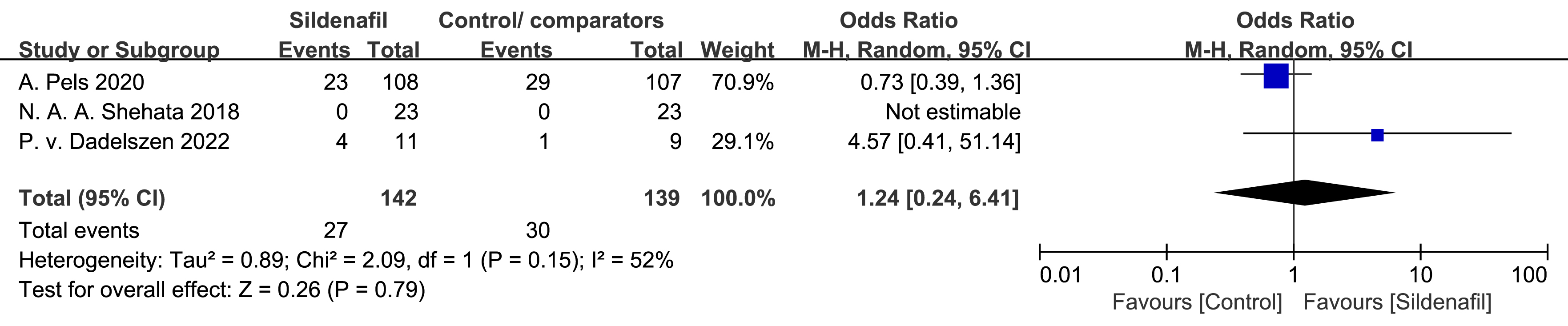

Supplement: Supplementary file 2 [file Image9.JPEG]

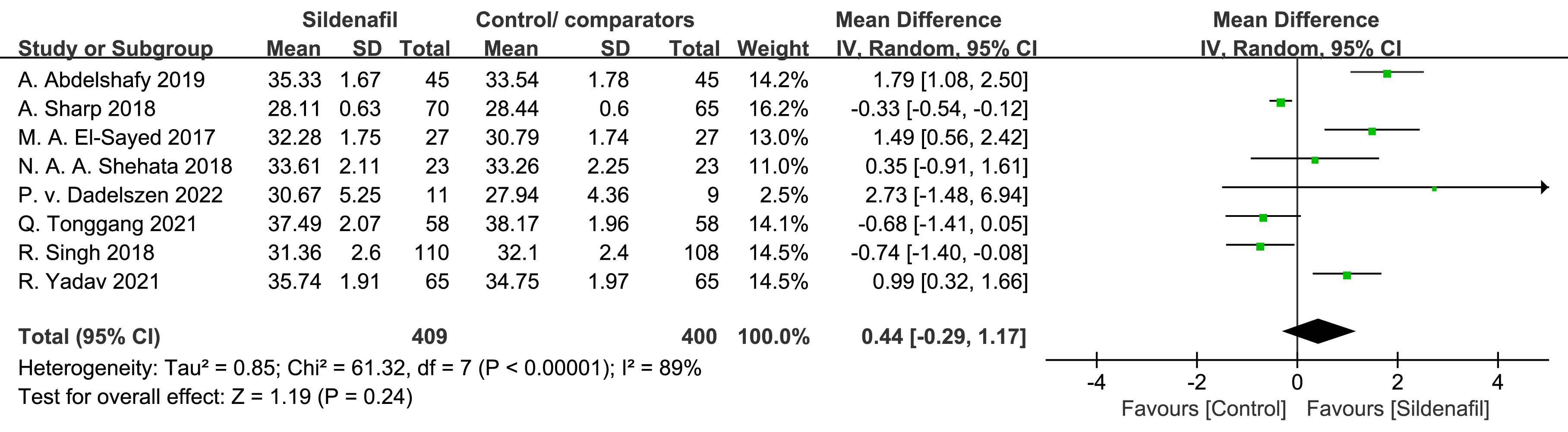

Supplement: Supplementary file 3 [file Image1.JPEG]

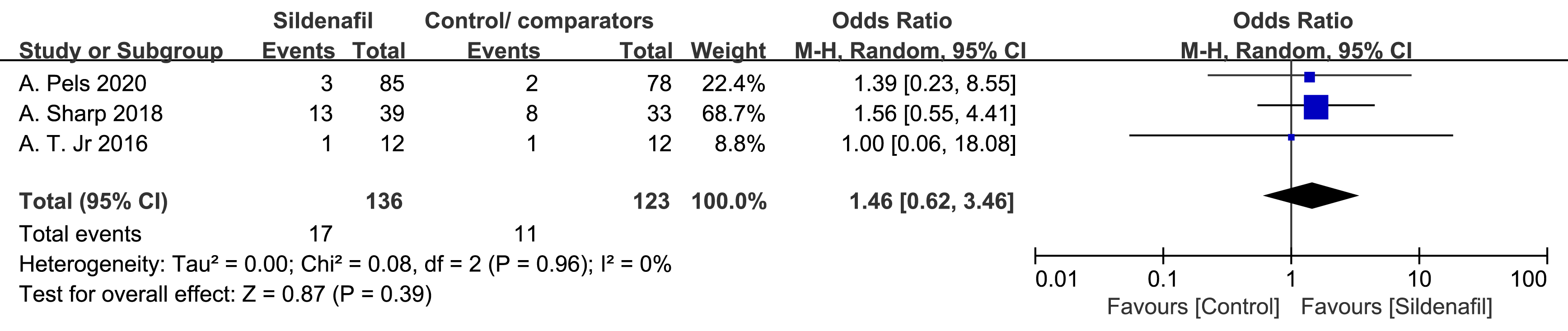

Supplement: Supplementary file 4 [file Image4.JPEG]

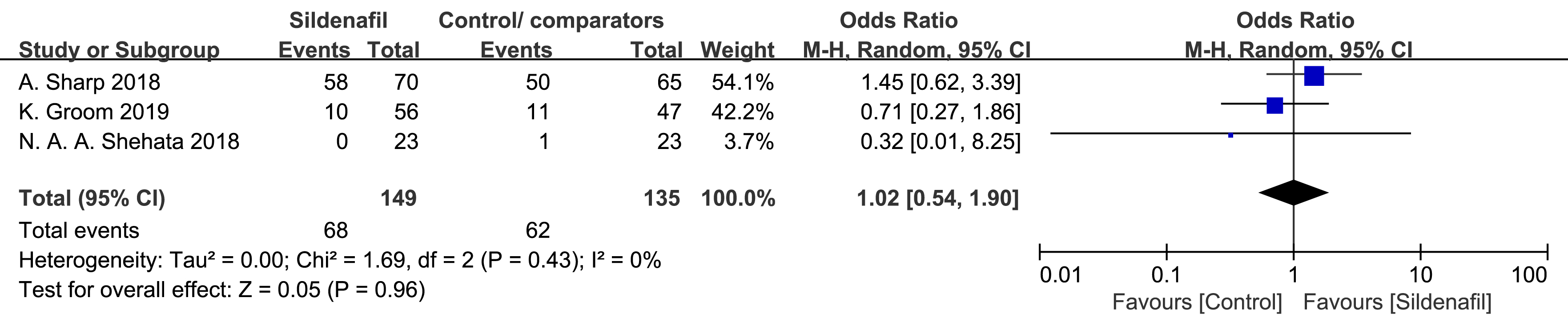

Supplement: Supplementary file 5 [file Image7.JPEG]

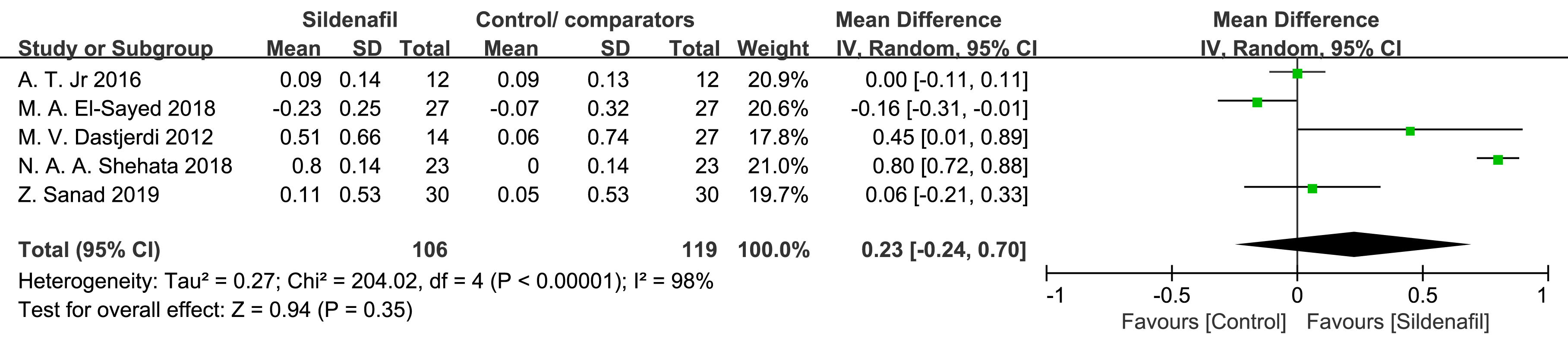

Supplement: Supplementary file 6 [file Image2.JPEG]

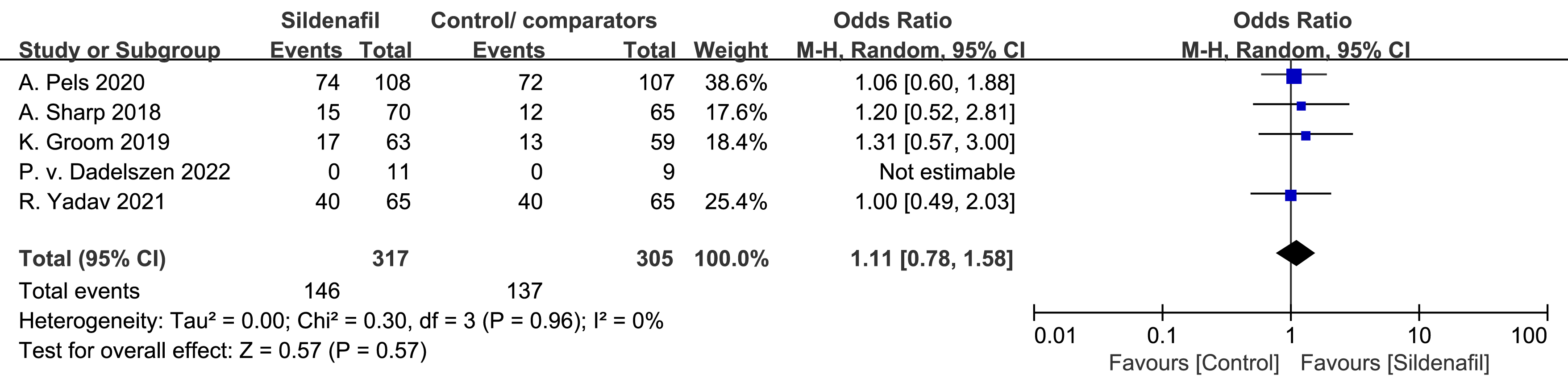

Supplement: Supplementary file 7 [file Image5.JPEG]

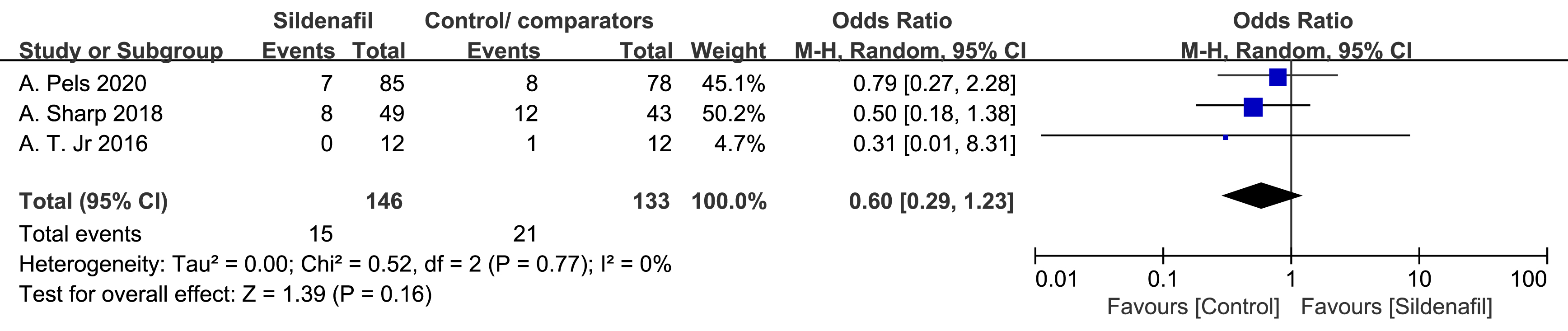

Supplement: Supplementary file 8 [file Image10.JPEG]

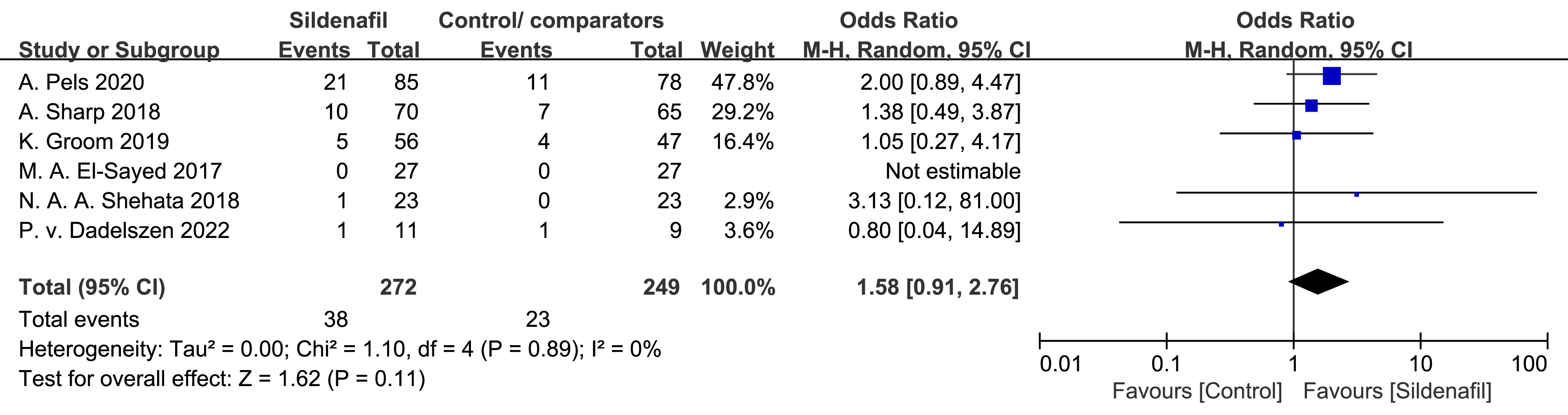

Supplement: Supplementary file 11 [file Image8.JPEG]

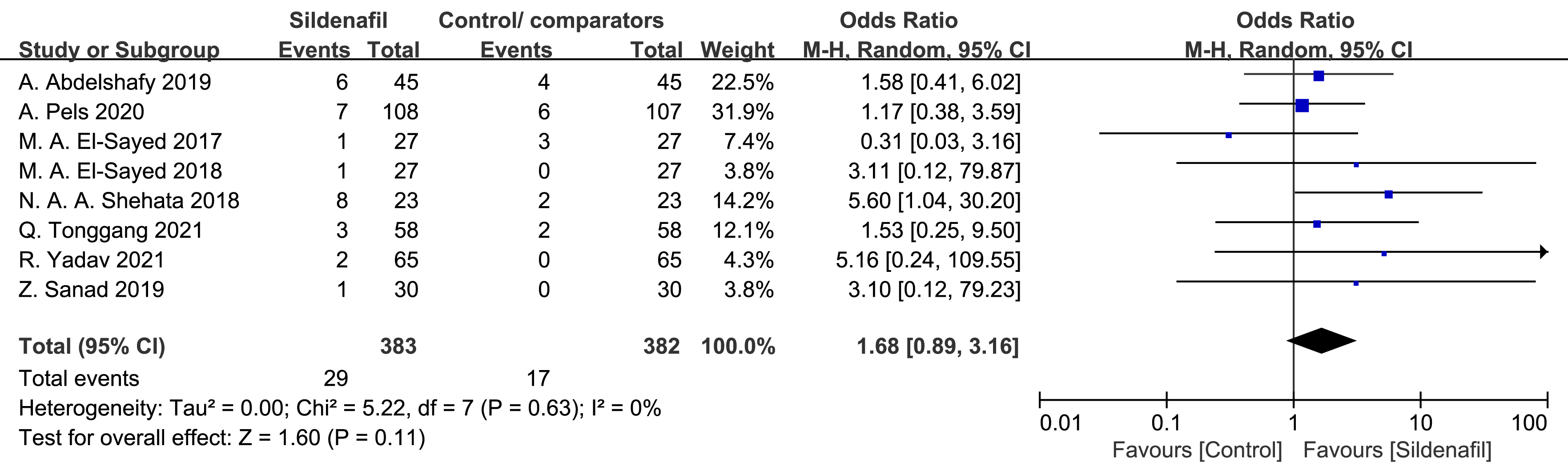

Supplement: Supplementary file 12 [file Image6.JPEG]
