# Supplementary material for: Safety and efficacy of phosphodiesterase-5 (PDE-5) inhibitors in fetal growth restriction: a systematic literature review and meta-analysis
Source: J Pharm Pharm Sci. 2024 Aug 15;27:13206. doi: 10.3389/jpps.2024.13206 (PMC11357966; doi:10.3389/jpps.2024.13206)
Supplement: Supplementary file 9 [file DataSheet1.pdf]

Science Direct search strategy

PUBMED

Search No	Search terms	Hits
#1	((((fetal growth retardation[MeSH Terms]) OR (Intrauterine Growth Retardation[Title/Abstract])) OR (Intrauterine Growth Restriction[Title/Abstract])) OR (Fetal Growth Restriction[Title/Abstract])) OR (Growth Retardation, Intrauterine[Title/Abstract])	28303
#2	(((((((((((((((Phosphodiesterase 5 Inhibitors[MeSH Terms]) OR (Inhibitors, Phosphodiesterase 5[Title/Abstract])) OR (PDE5 Inhibitor[Title/Abstract])) OR (Inhibitor, PDE5[Title/Abstract])) OR (Phosphodiesterase 5 Inhibitor[Title/Abstract])) OR (Inhibitor, Phosphodiesterase[Title/Abstract])) OR (Inhibitor, Phosphodiesterase 5[Title/Abstract])) OR (PDE-5 Inhibitor[Title/Abstract])) OR (Inhibitor, PDE-5[Title/Abstract])) OR (PDE 5 Inhibitor[Title/Abstract])) OR (PDE-5 Inhibitors[Title/Abstract])) OR (Inhibitors, PDE-5[Title/Abstract])) OR (PDE 5 Inhibitors[Title/Abstract])) OR (PDE5 Inhibitors[Title/Abstract])) OR (Inhibitors, PDE5[Title/Abstract])) OR (Phosphodiesterase Type 5 Inhibitors[Title/Abstract])) OR (Phosphodiesterase Type 5 Inhibitor[Title/Abstract])	7343
#3	(((((((((((((Sildenafil Citrate[MeSH Terms]) OR (Citrate, Sildenafil[Title/Abstract])) OR (Revatio[Title/Abstract])) OR (Sildenafil[Title/Abstract])) OR (Homosildenafil[Title/Abstract])) OR (Hydroxyhomosildenafil[Title/Abstract])) OR (Viagra[Title/Abstract])) OR (Acetildenafil[Title/Abstract])) OR (Sildenafil Lactate[Title/Abstract])) OR (Lactate, Sildenafil[Title/Abstract])) OR (Sildenafil Nitrate[Title/Abstract])) OR (Nitrate, Sildenafil[Title/Abstract])) OR (Desmethyl Sildenafil[Title/Abstract])) OR (Sildenafil, Desmethyl[Title/Abstract])) OR (Desmethylsildenafil[Title/Abstract])	8866
#4	((((((((((((((((((((Tadalafil[MeSH Terms])) OR (Cialis[Title/Abstract])) OR (Vardenafil Dihydrochloride[Title/Abstract])) OR (Dihydrochloride, Vardenafil[Title/Abstract])) OR (Vardenafil Hydrochloride Anhydrous[Title/Abstract])) OR (Anhydrous, Vardenafil Hydrochloride[Title/Abstract])) OR (Hydrochloride Anhydrous, Vardenafil[Title/Abstract])) OR (Vardenafil[Title/Abstract])) OR (Levitra[Title/Abstract])) OR (Vardenafil Hydrochloride Trihydrate[Title/Abstract])) OR (Vardenafil Hydrochloride Trihydrate[Title/Abstract])) OR (Trihydrate, Vardenafil Hydrochloride[Title/Abstract])) OR (Vardenafil Hydrochloride[Title/Abstract])) OR (Hydrochloride, Vardenafil[Title/Abstract])) OR (avanafil[Title/Abstract])) OR (Stendra[Title/Abstract])) OR (Udenafil[Title/Abstract])) OR (Zidena[Title/Abstract])) OR (Aildenafil[Title/Abstract])) OR	2975

	(mirodenafil[Title/Abstract])	
#5	#2 OR #3 OR #4	13995
#6	((((randomized controlled trial[Publication Type]) OR (controlled clinical trial[Title/Abstract])) OR (clinical trial[Title/Abstract])) OR (randomized[Title/Abstract])) OR (placebo[Title/Abstract])	1133320
#7	#1 AND #5 AND #6	40

WEB OF SCIENCE

Search No	Search terms	Hits
#1	TS=(fetal growth retardation OR Intrauterine Growth Retardation OR Intrauterine Growth Restriction OR Fetal Growth Restriction OR Growth Retardation, Intrauterine)	50538
#2	TS=(Phosphodiesterase 5 Inhibitors OR Inhibitors, Phosphodiesterase 5 OR PDE5 Inhibitor OR Inhibitor, PDE5 OR Phosphodiesterase 5 Inhibitor OR 5 Inhibitor, Phosphodiesterase OR Inhibitor, Phosphodiesterase 5 OR PDE-5 Inhibitor OR Inhibitor, PDE-5 OR PDE 5 Inhibitor OR PDE-5 Inhibitors OR Inhibitors, PDE-5 OR PDE 5 Inhibitors OR PDE5 Inhibitors OR Inhibitors, PDE5 OR Phosphodiesterase Type 5 Inhibitors OR Phosphodiesterase Type 5 Inhibitor OR Sildenafil Citrate OR Citrate, Sildenafil OR Revatio OR Sildenafil OR Homosildenafil OR Hydroxyhomosildenafil OR Viagra OR Acetildenafil OR Sildenafil Lactate OR Lactate, Sildenafil OR Sildenafil Nitrate OR Nitrate, Sildenafil OR Desmethyl Sildenafil OR Sildenafil, Desmethyl OR Desmethylsildenafil OR Tadalafil OR Cialis OR Vardenafil Dihydrochloride OR Dihydrochloride, Vardenafil OR Vardenafil Hydrochloride Anhydrous OR Anhydrous, Vardenafil Hydrochloride OR Hydrochloride Anhydrous, Vardenafil OR Vardenafil OR Levitra OR Vardenafil Hydrochloride Trihydrate OR Hydrochloride Trihydrate, Vardenafil OR Trihydrate, Vardenafil Hydrochloride OR Vardenafil Hydrochloride OR Hydrochloride, Vardenafil OR avanafil OR Stendra OR Udenafil Zidena OR Aildenafil OR mirodenafil)	23972
#3	TS=(controlled clinical trial OR randomized controlled trial OR clinical trial OR randomized OR placebo)	1667712
#4	#1 AND #2 AND #3	59
#5	#1 AND #2 AND #3 and Review Articles (Exclude – Document Types)	42

EMBASE

Search No	Search terms	Hits
#1	'intrauterine growth retardation'/exp OR 'fetal growth retardation':ab,ti OR 'intrauterine growth restriction':ab,ti OR 'fetal growth restriction':ab,ti OR 'growth retardation, intrauterine':ab,ti	63782
#2	'phosphodiesterase v inhibitor'/exp OR 'phosphodiesterase 5 inhibitors':ab,ti OR 'inhibitors, phosphodiesterase 5':ab,ti OR 'pde5 inhibitor':ab,ti OR 'inhibitor, pde5':ab,ti OR 'phosphodiesterase 5	37294

	inhibitor':ab,ti OR '5 inhibitor, phosphodiesterase':ab,ti OR 'inhibitor, phosphodiesterase 5':ab,ti OR 'pde-5 inhibitor':ab,ti OR 'inhibitor, pde-5':ab,ti OR 'pde 5 inhibitor':ab,ti OR 'pde-5 inhibitors':ab,ti OR 'inhibitors, pde-5':ab,ti OR 'pde 5 inhibitors':ab,ti OR 'pde5 inhibitors':ab,ti OR 'inhibitors, pde5':ab,ti OR 'phosphodiesterase type 5 inhibitors':ab,ti OR 'phosphodiesterase type 5 inhibitor':ab,ti OR 'sildenafil'/exp OR 'sildenafil citrate':ab,ti OR 'citrate, sildenafil':ab,ti OR 'revatio':ab,ti OR 'homosildenafil':ab,ti OR 'hydroxyhomosildenafil':ab,ti OR 'viagra':ab,ti OR 'acetildenafil':ab,ti OR 'sildenafil lactate':ab,ti OR 'lactate, sildenafil':ab,ti OR 'sildenafil nitrate':ab,ti OR 'nitrate, sildenafil':ab,ti OR 'desmethyl sildenafil':ab,ti OR 'sildenafil, desmethyl':ab,ti OR 'desmethylsildenafil':ab,ti OR 'tadalafil'/exp OR 'cialis':ab,ti OR 'vardenafil'/exp OR 'vardenafil dihydrochloride':ab,ti OR 'dihydrochloride, vardenafil':ab,ti OR 'vardenafil hydrochloride anhydrous':ab,ti OR 'anhydrous, vardenafil hydrochloride':ab,ti OR 'hydrochloride anhydrous, vardenafil':ab,ti OR 'levitra':ab,ti OR 'vardenafil hydrochloride trihydrate':ab,ti OR 'hydrochloride trihydrate, vardenafil':ab,ti OR 'trihydrate, vardenafil hydrochloride':ab,ti OR 'vardenafil hydrochloride':ab,ti OR 'hydrochloride, vardenafil':ab,ti OR 'avanafil'/exp OR 'stendra':ab,ti OR 'udenafil'/exp OR 'udenafil':ab,ti OR 'zidena':ab,ti OR 'aildenafil':ab,ti OR 'mirodenafil'/exp	
#3	'controlled clinical trial':ab,ti OR 'randomized controlled trial':ab,ti OR 'clinical trial':ab,ti OR 'randomized':ab,ti OR 'placebo':ab,ti	1324052
#4	#1 AND #2 AND #3	61

COCHRANE

Search No	Search terms	Hits
#1	MeSH descriptor: [Fetal Growth Retardation] explode all trees	546
#2	(Intrauterine Growth Retardation):ti,ab,kw OR (Intrauterine Growth Restriction):ti,ab,kw OR (Fetal Growth Restriction):ti,ab,kw OR (Growth Retardation, Intrauterine):ti,ab,kw	1454
#3	#1 or #2	1627
#4	MeSH descriptor: [Phosphodiesterase 5 Inhibitors] explode all trees	465
#5	MeSH descriptor: [Tadalafil] explode all trees	542
#6	MeSH descriptor: [Sildenafil Citrate] explode all trees	1098
#7	MeSH descriptor: [Vardenafil Dihydrochloride] explode all trees	192
#8	(Inhibitors, Phosphodiesterase 5):ti,ab,kw OR (PDE5 Inhibitor):ti,ab,kw OR (Inhibitor, PDE5):ti,ab,kw OR (Phosphodiesterase 5 Inhibitor):ti,ab,kw OR (5 Inhibitor, Phosphodiesterase):ti,ab,kw OR (Inhibitor, Phosphodiesterase 5):ti,ab,kw OR (PDE-5 Inhibitor):ti,ab,kw OR (Inhibitor, PDE-5):ti,ab,kw OR (PDE 5 Inhibitor):ti,ab,kw OR (PDE-5 Inhibitors):ti,ab,kw OR (Inhibitors, PDE-5):ti,ab,kw OR (PDE 5 Inhibitors):ti,ab,kw OR (PDE5 Inhibitors):ti,ab,kw OR (Inhibitors,	2407

	PDE5):ti,ab,kw OR (Phosphodiesterase Type 5 Inhibitors):ti,ab,kw OR (Phosphodiesterase Type 5 Inhibitor):ti,ab,kw	
#9	(Citrate, Sildenafil):ti,ab,kw OR (Revatio):ti,ab,kw OR (Sildenafil):ti,ab,kw OR (Homosildenafil):ti,ab,kw OR (Hydroxyhomosildenafil):ti,ab,kw OR (Viagra):ti,ab,kw OR (Acetildenafil):ti,ab,kw OR (Sildenafil Lactate):ti,ab,kw OR (Lactate, Sildenafil):ti,ab,kw OR (Sildenafil Nitrate):ti,ab,kw OR (Nitrate, Sildenafil):ti,ab,kw OR (Desmethyl Sildenafil):ti,ab,kw OR (Sildenafil, Desmethyl):ti,ab,kw OR (Desmethylsildenafil):ti,ab,kw	2282
#10	(Cialis):ti,ab,kw	80
#11	(Dihydrochloride, Vardenafil):ti,ab,kw OR (Vardenafil Hydrochloride Anhydrous):ti,ab,kw OR (Anhydrous, Vardenafil Hydrochloride):ti,ab,kw OR (Hydrochloride Anhydrous, Vardenafil):ti,ab,kw OR (Vardenafil):ti,ab,kw OR (Levitra):ti,ab,kw OR (Vardenafil Hydrochloride Trihydrate):ti,ab,kw OR (Hydrochloride Trihydrate, Vardenafil):ti,ab,kw OR (Trihydrate, Vardenafil Hydrochloride):ti,ab,kw OR (Vardenafil Hydrochloride):ti,ab,kw OR (Hydrochloride, Vardenafil):ti,ab,kw OR (avanafil):ti,ab,kw OR (Stendra):ti,ab,kw OR (Udenafil):ti,ab,kw OR (Zidena):ti,ab,kw OR (Aildenafil):ti,ab,kw OR (mirodenafil):ti,ab,kw	526
#12	#4 OR #5 OR #6 OR #7 OR #8 OR #9 OR #10 OR #11	4443
#13	(controlled clinical trial):ti,ab,kw OR (randomized controlled trial):ti,ab,kw OR (clinical trial):ti,ab,kw OR (randomized):ti,ab,kw OR (placebo):ti,ab,kw	1327659
#14	#3 AND #12 AND #13	59

MEDLINE

Search No	Search terms	Hits
S1	(SU fetal growth retardation OR SU Intrauterine Growth Retardation OR SU Growth Retardation, Intrauterine OR SU Intrauterine Growth Restriction OR SU Fetal Growth Restriction)	2506
S2	(SU Phosphodiesterase 5 Inhibitors OR SU Inhibitors, Phosphodiesterase 5 OR SU PDE5 Inhibitor OR SU Inhibitor, PDE5 OR SU Phosphodiesterase 5 Inhibitor OR SU 5 Inhibitor, Phosphodiesterase OR SU Inhibitor, Phosphodiesterase 5 OR SU PDE-5 Inhibitor OR SU Inhibitor, PDE-5 OR SU PDE 5 Inhibitor OR SU PDE-5 Inhibitors OR SU Inhibitors, PDE-5 OR SU PDE 5 Inhibitors OR SU PDE5 Inhibitors OR SU Inhibitors, PDE5 OR SU Phosphodiesterase Type 5 Inhibitors OR SU Phosphodiesterase Type 5 Inhibitor OR SU Sildenafil Citrate OR SU Citrate, Sildenafil OR SU Revatio OR SU Sildenafil OR SU Homosildenafil OR SU Hydroxyhomosildenafil OR SU Viagra OR SU Acetildenafil OR SU Sildenafil Lactate OR SU Lactate, Sildenafil OR SU Sildenafil Nitrate OR SU Nitrate, Sildenafil OR SU Desmethyl Sildenafil OR SU Sildenafil, Desmethyl OR SU Desmethylsildenafil OR SU Tadalafil OR SU Cialis OR SU Vardenafil Dihydrochloride OR SU Dihydrochloride, Vardenafil OR	9619

	SU Vardenafil Hydrochloride Anhydrous OR SU Anhydrous, Vardenafil Hydrochloride OR SU Hydrochloride Anhydrous, Vardenafil OR SU Vardenafil OR SU Levitra OR SU Vardenafil Hydrochloride Trihydrate OR SU Hydrochloride Trihydrate, Vardenafil OR SU Trihydrate, Vardenafil Hydrochloride OR SU Vardenafil Hydrochloride OR SU Hydrochloride, Vardenafil OR SU avanafil OR SU Stendra OR SU Udenafil OR SU Zidena OR SU Aildenafil OR SU mirodenafil)	
S3	(PT randomized controlled trial OR AB controlled clinical trial OR AB clinical trial OR AB randomized OR AB placebo OR TI controlled clinical trial OR TI clinical trial OR TI randomized OR TI placebo)	1,242,514
S4	S1 AND S2 AND S3	17

CNKI

Search No	Search terms	Hits
#1	(SU='胎儿生长迟缓' OR SU='发育迟缓，宫内' OR SU='宫内生长迟缓' OR SU='胎儿生长受限' OR SU='胎儿宫内生长受限' OR SU='宫内生长受限' OR SU='生长受限')	19288
#2	(SU='磷酸二酯酶 5 抑制剂' OR SU='5 型磷酸二酯酶抑制剂' OR SU=' PDE5 抑制剂' OR SU='枸橼酸西地那非' OR SU='西地那非' OR SU='万艾可' OR SU='他达拉非' OR SU='西力士' OR SU='伐地那非' OR SU=' 阿伐那非' OR SU='乌地那非' OR SU='米罗那非')	3644
#3	(SU='临床对照试验' OR SU='随机对照试验' OR SU='临床试验' OR SU='随机' OR SU='安慰剂')	369386
#4	#1 AND #2 AND #3	25

CBM

Search No	Search terms	Hits
#1	("胎儿生长迟缓"[不加权:扩展]) OR "发育迟缓，宫内" OR "宫内生长迟缓" OR "胎儿生长受限" OR "胎儿宫内生长受限" OR "宫内生长受限" OR "生长受限"	6889
#2	("磷酸二酯酶 5 抑制剂"[不加权:扩展]) OR ("枸橼酸西地那非"[不加权:扩展]) OR ("他达拉非"[不加权:扩展]) OR "5 型磷酸二酯酶抑制剂" OR " PDE5 抑制剂" OR "西地那非" OR "万艾可" OR "西力士" OR "伐地那非" OR "阿伐那非" OR "乌地那非" OR "米罗那非"	2868
#3	("临床对照试验"[不加权:扩展]) OR ("随机对照试验"[不加权:扩展]) OR ("临床试验"[不加权:扩展]) OR ("安慰剂"[不加权:扩展]) OR "随机"	1768430
#4	#1 AND #2 AND #3	5

WANFANG DETA

Search No	Search terms	Hits
#1	(TI=(”胎儿生长迟缓” OR “发育迟缓，宫内” OR “宫内生长迟缓” OR “胎儿生长受限” OR “胎儿宫内生长受限” OR “宫内生长受限” OR “生长受限”) OR AB=(”胎儿生长迟缓” OR “发育迟缓，宫内” OR “宫内生长迟缓” OR “胎儿生长受限” OR “胎儿宫内生长受限” OR “宫内生长受限” OR “生长受限”))	31
#2	(TI=(”磷酸二酯酶 5 抑制剂” OR “5 型磷酸二酯酶抑制剂” OR “ PDE5 抑制剂” OR “枸橼酸西地那非” OR “西地那非” OR “万艾可” OR “他达拉非” OR “西力士” OR “伐地那非” OR “阿伐那非” OR “乌地那非” OR “米罗那非) OR AB=(”磷酸二酯酶 5 抑制剂” OR “5 型磷酸二酯酶抑制剂” OR “ PDE5 抑制剂” OR “枸橼酸西地那非” OR “西地那非” OR “万艾可” OR “他达拉非” OR “西力士” OR “伐地那非” OR “阿伐那非” OR “乌地那非” OR “米罗那非))	14
#3	(TI=(”临床对照试验” OR “随机对照试验” OR “临床试验” OR “随机” OR “安慰剂”) OR AB=(”临床对照试验” OR “随机对照试验” OR “临床试验” OR “随机” OR “安慰剂”))	378
#4	#1 AND #2 AND #3	4
